# Exploring Huntington’s Disease Diagnosis via Artificial Intelligence Models: A Comprehensive Review

**DOI:** 10.3390/diagnostics13233592

**Published:** 2023-12-03

**Authors:** Sowmiyalakshmi Ganesh, Thillai Chithambaram, Nadesh Ramu Krishnan, Durai Raj Vincent, Jayakumar Kaliappan, Kathiravan Srinivasan

**Affiliations:** 1 School of Computer Science and Engineering, Vellore Institute of Technology, Vellore 632014, Tamil Nadu, India; sowmiyalakshmi.g2020@vitstudent.ac.in (S.G.); thillaichithambaram.m2020@vitstudent.ac.in (T.C.); jayakumar.k@vit.ac.in (J.K.); 2 School of Computer Science Engineering and Information Systems, Vellore Institute of Technology, Vellore 632014, Tamil Nadu, India; rknadesh@vit.ac.in

**Keywords:** Huntington’s disease, Artificial Intelligence, machine learning, deep learning, diagnosis

## Abstract

Huntington’s Disease (HD) is a devastating neurodegenerative disorder characterized by progressive motor dysfunction, cognitive impairment, and psychiatric symptoms. The early and accurate diagnosis of HD is crucial for effective intervention and patient care. This comprehensive review provides a comprehensive overview of the utilization of Artificial Intelligence (AI) powered algorithms in the diagnosis of HD. This review systematically analyses the existing literature to identify key trends, methodologies, and challenges in this emerging field. It also highlights the potential of ML and DL approaches in automating HD diagnosis through the analysis of clinical, genetic, and neuroimaging data. This review also discusses the limitations and ethical considerations associated with these models and suggests future research directions aimed at improving the early detection and management of Huntington’s disease. It also serves as a valuable resource for researchers, clinicians, and healthcare professionals interested in the intersection of machine learning and neurodegenerative disease diagnosis.

## 1. Introduction

Huntington’s disease is a profoundly impactful neurodegenerative disorder [1] that not only affects individuals but also casts a long shadow over their families [2]. It represents a complex clinical picture, marked by the inexorable progression of motor dysfunction, cognitive decline, and psychiatric symptoms, ultimately culminating in profound disability and a tragically shortened lifespan [3]. The gravity of HD has sparked growing concern among the medical and research communities worldwide, triggering multifaceted efforts to not only unravel its etiological and pathophysiological intricacies but also to pioneer advancements in its early detection and management [4]. Research in other neurodegenerative diseases such as Alzheimer’s [5,6,7] and Parkinson’s [8] has similarly aimed to decode their intricate mechanisms, leading to strides in understanding their pathology and paving the way for potential treatment breakthroughs.

Recent epidemiological studies have illuminated the prevalence and incidence of HD, revealing the stark reality of this disease. The pooled incidence of HD, as reported across various populations, has been estimated at 0.48 cases per 100,000 person-years (95% CI, 0.33–0.63). This statistic underscores the challenging nature of diagnosing HD, especially in its nascent stages, given its relative rarity. Furthermore, a continent-based analysis of these figures uncovers marked disparities in the incidence of HD, with Europe and North America experiencing considerably higher rates compared to Asia. Beyond incidence, comprehending the prevalence of HD is essential for effective healthcare planning and resource allocation. The compiled prevalence of HD stands at 4.88 per 100,000 (95% CI, 3.38–7.06) [9], shedding light on the overall burden of the disease within populations. These prevalence figures not only serve as an alarming reminder of the global health concern that HD represents but also emphasize the urgent need for concerted efforts to enhance its diagnosis, treatment, and support systems for affected individuals and their families.

The pathological progression of Huntington’s disease (HD) remains elusive, drawing attention from varied research domains. The work [10] explores motor speech patterns, reflecting the motor involvement in HD. The ref. [11] delves into speech biomarkers across HD stages, emphasizing the continuum from pre-symptomatic to early manifestation. An another article [12] contributes insights on Huntington’s multifaceted role, spanning neurodevelopment to neurodegeneration. Understanding synaptic loss, the ref. [13] and another study in Nature Medicine [14] highlight the early involvement of microglia, complement activation, and innate immune mechanisms in corticostriatal synapse decline. The study [15] offers perspectives on the toxic effects of mutant Huntington. This multifaceted exploration underscores the complexity of HD’s pathological cascade, spanning molecular, synaptic, and clinical dimensions.

This comprehensive review embarks on an exploration of a highly promising avenue for augmenting the early diagnosis of HD—the utilization of machine learning (ML) and deep learning (DL) models [16,17]. The convergence of cutting-edge technology with the realm of clinical medicine offers an exciting prospect: the ability to identify HD at its incipient stages, potentially enabling more effective interventions. Through a methodical examination of the existing literature on ML and DL models for HD diagnosis, it aspires to provide invaluable insights into the potential of these techniques and the complex challenges they pose. One of the key aspects this review will explore is the wide range of data sources that can be harnessed to train and validate ML and DL models for HD diagnosis. From medical imaging data such as MRI and CT scans to genetic markers and clinical records, the breadth of available information offers an opportunity to develop comprehensive diagnostic models. The integration of multi-modal data and the development of hybrid models that combine different data types could be a significant focus of the investigation. This review will address the challenges and ethical considerations associated with the implementation of AI-powered models in clinical practice. Issues such as data privacy, model interpretability, and the need for robust validation and regulatory approval will be explored. Additionally, the review will consider the potential biases that can arise in ML and DL approaches and strategies to mitigate them, ensuring that these technologies are deployed in a fair and equitable manner. The importance of early diagnosis in HD cannot be overstated, as it paves the way for timely interventions that can potentially slow down the progression of this devastating neurodegenerative disorder. By synthesizing existing knowledge and offering critical insights, it seeks to inspire further research and innovation in this field, ultimately advancing our ability to diagnose and treat HD at its earliest stages, potentially improving the quality of life for affected individuals and their families. This review, therefore, stands as an indispensable resource for researchers, clinicians, and healthcare professionals alike, who are keen to harness the formidable power of artificial intelligence to make meaningful strides in improving the lives of those affected by Huntington’s disease.

This work explores the potential of artificial intelligence to improve the diagnosis of Huntington’s disease. It offers an extensive examination of AI-powered techniques that have been applied to this condition, and assesses their effectiveness. The motive behind this paper is to bridge the gap between AI and HD by demonstrating the usefulness of these technologies in this context. The paper aims to provide a resource for researchers and practitioners interested in using AI to improve the diagnosis and treatment of HD. The significant contributions of this study are summarized as follows:

- This paper represents the pioneering effort to comprehensively compare the effectiveness of AI powered approaches for HD diagnosis, providing a critical synthesis of their respective strengths and potential clinical applications.
- This study presents a thorough examination of the various AI powered techniques that have been applied to the diagnosis of Huntington’s disease. It includes an overview of different ML and DL methodologies and how they have been used in this context.
- This review addresses current challenges and identifies future research opportunities in the use of ML and DL for Huntington’s disease diagnosis. It aims to provide information and inspiration for aspiring researchers and enthusiasts interested in pursuing this topic.

### Arrangement of This Review

**Figure 1:**
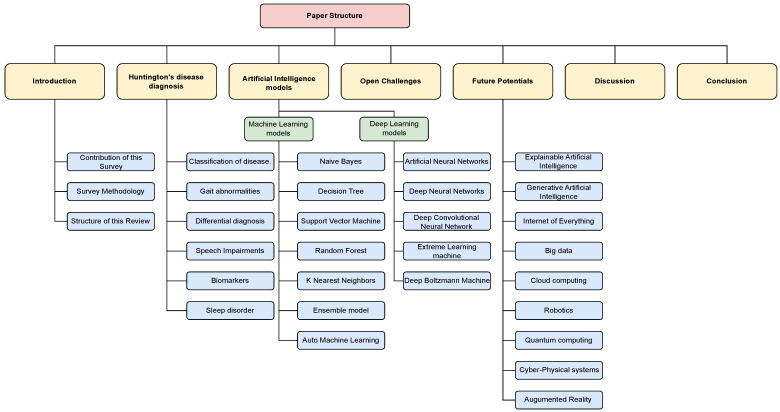
Structure of this review.

Figure 1 comprehensively outlines the structure and organization of the paper. The sections covered in the paper include the Introduction, Survey Methodology, Huntington’s Disease Diagnosis, Diagnosis of Huntington’s disease via AI-powered models, Open Challenges, Future Research Directions, and Conclusion.

## 2. Survey Methodology

**Figure 2:**
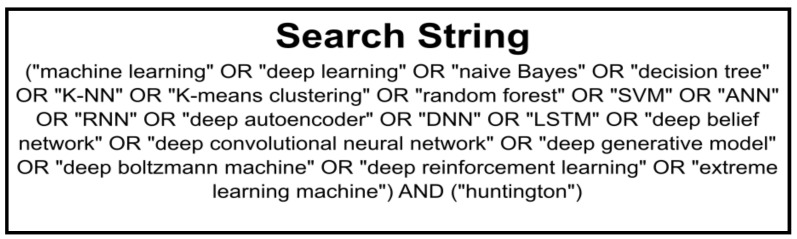
Search String for querying from the database.

The papers incorporated in this review were curated utilizing the “Preferred Reporting Items for Systematic reviews and Meta-Analyses extension for Scoping Reviews (PRISMA-ScR)” methodology [18]. Additionally, the selection process was guided by the designated search string, as illustrated in Figure 2.

### 2.1. Search Strategy, Databases, and Screening Criteria

This study conducted a search for articles on developing metaheuristic algorithms for Huntington’s disease diagnosis published between January 2005 and July 2023 on various databases, including ACM Digital Library, IEEE Xplore, Springer, ScienceDirect, and Google Scholar. The search for relevant articles for this study involved using keywords including “Huntington’s disease”, “Huntington’s disease diagnosis”, “neurodegenerative diseases”, “deep learning methods”, “neural networks”, “machine learning”, and “artificial intelligence”. This search yielded over 253 articles. This study encompasses an examination of articles concerning the application of machine learning and deep learning approaches for diagnosing Huntington’s disease, specifically focusing on publications in the English language from January 2005 to August 2023. Its aim is to offer a summary of recent advancements in this domain while pinpointing potential directions for future research. The goal is to contribute to the advancement of knowledge in this area and to guide future studies on the use of AI for HD diagnosis. This survey only included articles published in English and after January 2005. It excluded case studies, analyses, reports, editorials, theses, doctoral dissertations, and analyses published in other languages or prior to January 2005.

### 2.2. Quality Assessment

Four trained assessors (S.G., T.C., P.M.D.R.V., and K.S.) conducted the search. In the case of disagreements or discrepancies, a consensus was reached among the assessors. Additionally, two other assessors (N.R.K. and J.K.) were consulted during the disagreements or discrepancies. The titles and abstracts of previously collected articles were meticulously examined, excluding any studies deemed irrelevant. Relevant articles were thoroughly reviewed, analyzing their complete texts, documenting the findings, and identifying any similar studies that met the inclusion/exclusion criteria. The selected articles were evaluated and approved by an anonymous clinical physician.

### 2.3. Results

**Identification:** During the identification phase, a comprehensive search across various databases yielded 247 relevant articles, while an additional six articles were obtained from other sources, resulting in a total of 253 articles identified for this comprehensive review.
**Screening:** In the screening phase, a meticulous process that included the removal of duplicates led to a selection of 178 unique articles. Subsequently, following title screening, 79 articles were excluded, and an additional 47 were eliminated after the abstract screening. In total, 126 articles were removed during the screening process, leaving a focused set of articles for further review and analysis.
**Eligibility:** During the eligibility phase, a rigorous assessment revealed that 19 articles were unrelated to Huntington’s disease, and in 11 articles, no Machine Learning (ML) or Deep Learning (DL) models were utilized. Consequently, a total of 30 papers were excluded from the review, ensuring that the remaining articles are directly relevant to the exploration of Huntington’s disease diagnosis via AI models.
**Included:** In the inclusion phase, after careful evaluation, a final selection of 22 articles met the criteria for inclusion in the comprehensive review.

**Figure 3:**
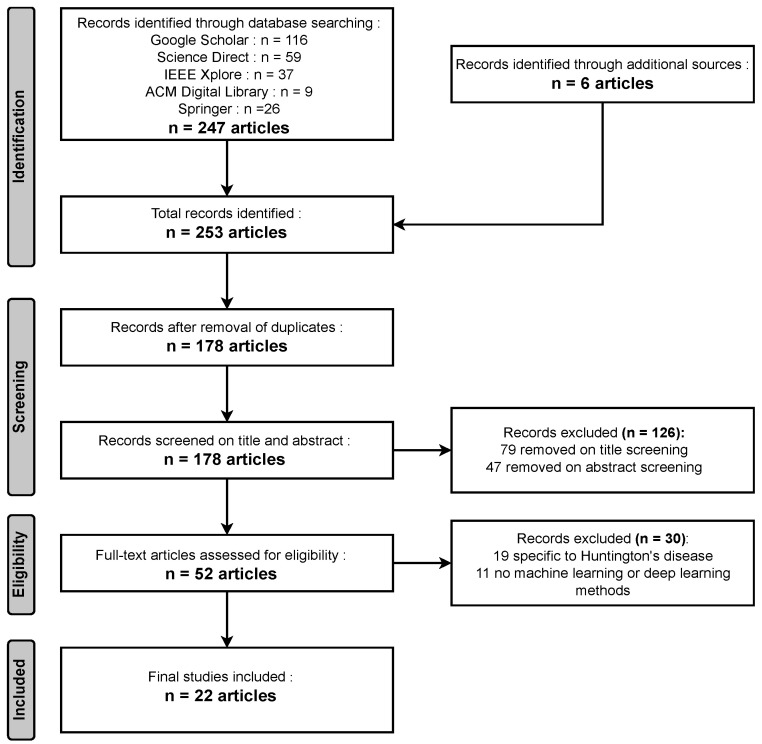
PRISMA-ScR flow chart for the inclusion process in this scoping review.

Figure 3 illustrates that adhering to the inclusion and exclusion criteria, a total of 22 papers were obtained as outcomes.

## 3. Background of Huntington’s Disease Diagnosis

Huntington’s disease is a devastating neurodegenerative disorder characterized by a progressive deterioration of motor function, cognitive decline, and psychiatric disturbances. This condition arises due to a mutation in the HTT gene that follows an autosomal dominant inheritance pattern, resulting in the abnormal expansion of CAG (Cytosine, Adenine, Guanine) repeats within the gene. The longer the CAG repeats, the earlier the onset and the more severe the symptoms [19]. HD typically manifests in mid-adulthood, with a wide range of age at onset, but it can also occur in childhood or late adulthood. Presently, Huntington’s disease lacks a cure, underscoring the significance of timely and precise diagnosis for patients and their families.

Diagnosing HD has historically relied on clinical assessments, including the observation of motor symptoms, psychiatric disturbances, and cognitive decline [20]. However, as the disease progresses, these symptoms become more evident, making it challenging to diagnose in the early stages. To address this challenge, advances in genetic testing have allowed for the direct identification of the CAG repeat expansion in the HTT gene, providing a definitive diagnosis. Genetic testing is highly accurate and has become the gold standard for HD diagnosis, enabling individuals at risk to undergo predictive testing before symptoms appear. Moreover, recent developments in neuroimaging, such as magnetic resonance imaging (MRI), have enabled clinicians to identify alterations in structure in the brain associated with HD, even before symptoms become apparent [21]. These imaging techniques can also help monitor disease progression and assess the effectiveness of potential treatments.

The field of HD diagnosis has witnessed substantial progress over the past few years with the emergence of biomarker research. Biomarkers are measurable indicators of disease processes and can include genetic, molecular, or neuroimaging markers [22]. Researchers are actively exploring various biomarkers to improve the accuracy of HD diagnosis and track disease progression. For instance, cerebrospinal fluid (CSF) analysis has revealed potential biomarkers related to neuroinflammation and neuronal damage [23]. Additionally, blood-based biomarkers and assays that measure specific proteins or metabolic changes are being investigated for their diagnostic potential [24]. Combining multiple biomarkers with clinical assessments and genetic testing holds promise for enhancing early and accurate HD diagnosis. Furthermore, ongoing research into disease-modifying therapies for HD underscores the importance of precise diagnosis, as early intervention may offer the best chance of slowing or halting disease progression.

### 3.1. Classification of Huntington’s Disease

HD can be classified into two main types depending on the age when symptoms first appear and disease progression: adult-onset HD and juvenile-onset HD [25]. These classifications help to differentiate between the timing of symptom onset and the progression of the disease, providing important insights into the clinical course of HD.

**Adult-Onset Huntington’s Disease:** Adult-onset HD is the more prevalent form of the condition, typically making its debut in individuals in the age range spanning from 30 to 50 years [26]. The disease’s signature motor symptoms, including chorea (involuntary, jerky movements), dystonia (sustained muscle contractions leading to abnormal postures), and gait abnormalities, are the primary early indicators. Cognitive and emotional manifestations, including memory deficits, mood fluctuations, and alterations in personality, tend to emerge as the disease progresses. While the rate of disease progression can vary, adult-onset HD generally advances more slowly than its juvenile-onset counterpart, although life expectancy is typically reduced, with an average survival of approximately 15 to 20 years following symptom onset.

**Juvenile-Onset Huntington’s Disease:** Juvenile-onset HD, although less common, presents a more aggressive form of the disorder. Symptoms typically arise in childhood or adolescence, often prior to reaching the age of twenty [27]. Juvenile-onset HD is marked by a more severe and rapidly progressing clinical course, with prominent motor symptoms such as chorea and dystonia. Cognitive decline and behavioral disturbances are also prevalent, frequently leading to significant impairments in school performance and social functioning [28]. The disease course in juvenile-onset HD is marked by a swifter deterioration in motor and cognitive functions, resulting in greater disability and a shorter life expectancy. Some individuals may experience a rapid deterioration over just a few years, while others may have a somewhat more protracted course.

### 3.2. Gait Abnormalities

Gait abnormalities in HD are a prominent and debilitating characteristic of the ailment, significantly impacting the well-being and standard of living of affected individuals. These disturbances in walking patterns are often among the earliest motor symptoms to manifest and can provide valuable diagnostic insights in the clinical evaluation of HD patients. They typically present as a combination of chorea and dystonia. Chorea refers to involuntary, jerky, and rapid movements that affect various body parts, including the legs [29]. In the context of gait, chorea can lead to irregular and uncoordinated movements of the limbs, making it difficult for individuals to maintain a steady and balanced walking pattern. Dystonia, on the other hand, involves sustained muscle contractions that result in abnormal postures or twisting movements. In the context of gait abnormalities, dystonia can lead to the twisting of the feet or legs, causing individuals to walk with an uneven and irregular gait [30]. These involuntary movements and postural changes contribute to a distinctive, unsteady, and often unpredictable gait pattern in HD patients.

As the disease progresses, gait abnormalities tend to worsen, and individuals with HD may exhibit a wide-based gait, where their feet are placed far apart to maintain balance. This wide-based gait is an adaptive response to the loss of coordination and balance, as it provides a larger base of support [31]. However, it can also lead to instability and an increased risk of falls. Additionally, individuals with HD may experience freezing of gait, which refers to sudden and temporary episodes where they find it impossible to initiate or continue walking as if their feet are glued to the ground [32]. Freezing of gait can be particularly challenging and hazardous, as it can occur unexpectedly and increases the likelihood of experiencing falls and sustaining injuries. Moreover, gait disturbances in HD often coexist with other motor symptoms, such as bradykinesia (slowness of movement) and muscle weakness, further complicating the ability to walk smoothly and efficiently [33,34]. In the advanced stages of the disease, individuals with HD may require mobility aids, such as walkers or wheelchairs, to maintain their independence and safety.

### 3.3. Differential Diagnosis

The differential diagnosis of HD is a critical process in which other conditions that may cause similar symptoms are ruled out. This is important because the symptoms of HD, such as movement disorders, cognitive decline, and behavioural changes, can be caused by a wide range of conditions, and accurate diagnosis is essential for determining the appropriate treatment and management strategies for individual patients [35]. Some of the conditions that may be considered in the process of distinguishing between the different diagnoses of HD include:

**Parkinson’s disease:** This neurodegenerative disorder is characterised by tremors, rigidity, and difficulty with movement. While HD also involves movement disorders, the signs of Parkinson’s disease tend to be more symmetrical and respond well to medication, while HD symptoms are often asymmetrical and do not respond well to medication [36]. Recent advancements in gene prioritization strategies, as observed in the context of Parkinson’s disease (PD) and preeclampsia, underscore the significance of consensus strategies in unraveling the pathogenesis of neurodegenerative disorders. Studies such as [37,38] exemplify the potency of consensus strategies in prioritizing genes linked to disease etiology, akin to the endeavors witnessed in Huntington’s disease (HD) research. The study [37] proposed a consensus strategy for PD gene prioritization, merging multiple prioritization approaches, akin to ensemble models, to enhance the identification of genes relevant to PD pathogenesis. Similarly, the research [38] utilized a consensus strategy for preeclampsia, employing various prioritization strategies and bioinformatics analyses to identify crucial genes associated with the condition. These strategies, by amalgamating diverse data sources and methodologies, facilitate the identification of biologically significant genes, offering potential targets for understanding the pathogenesis of degenerative diseases such as HD and developing targeted therapeutics. Importantly, the success of such consensus strategies in identifying genes directly associated with the disease and those involved in relevant biological processes echoes the potential applicability of similar methodologies in HD research, augmenting efforts to comprehend the intricate mechanisms underlying the disease’s progression and potentially uncovering novel targets for intervention and therapy.
**Dementia:** This term refers to a decline in cognitive abilities, including memory, language, and problem-solving [39]. While HD also involves cognitive decline, dementia typically affects multiple cognitive domains, while HD primarily affects executive functions and problem-solving abilities [40].
**Schizophrenia:** This mental disorder is characterized by delusions, hallucinations, and other psychotic symptoms. While HD may also involve changes in behavior and cognition, schizophrenia typically involves more severe and persistent symptoms and does not involve movement disorders [41].
**Wilson’s disease:** This rare genetic disorder results from a shortage of the enzyme copper-transporting ATPase, this results in a buildup of copper in the body. Symptoms of Wilson’s disease may include movement disorders, cognitive decline, and behavioral changes, but typically also include liver dysfunction and other symptoms that are not seen in HD [42].
**Multiple sclerosis:** This autoimmune disorder is caused by harm inflicted upon the protective layer of myelin that encases nerve fibers within the central nervous system [43]. Symptoms of multiple sclerosis may include movement disorders, cognitive decline, and behavioral changes, but typically also include sensory symptoms, such as numbness and tingling, that are not seen in HD.

The process of differential diagnosis for HD typically encompasses a comprehensive assessment of the patient’s symptoms, their medical background, and the familial medical history as well as diagnostic tests such as imaging studies and genetic testing. In some cases, additional specialized testing may be necessary to confirm the diagnosis, such as a brain biopsy or lumbar puncture. The accurate and timely diagnosis of HD is essential for ensuring that patients receive the appropriate treatment and support. Early diagnosis can allow patients to make informed decisions about their care and can provide an opportunity for early intervention to slow the advancement of the illness and improve their standard of living. In addition, accurate diagnosis is crucial for enabling individuals undergoing treatment and their families to access support and resources, such as genetic counseling and specialized care, that can help them manage the challenges of HD. Ultimately, the goal of differential diagnosis for HD is to provide individuals undergoing treatment and their families with the data and assistance they need to navigate the complexities of this devastating disease.

### 3.4. Speech Impairments

Huntington’s disease is a condition characterized by the progressive degeneration of the central nervous system and is characterized by a wide range of clinical symptoms, including movement disorders, cognitive decline, and behavioral changes. Among the many symptoms of HD, speech impairments are common and can greatly influence the standard of living and the individual’s capacity to communicate with others [44]. The speech impairments seen in HD are typically caused by a combination of factors, including muscle weakness, difficulty with coordination and control, and cognitive decline. Some of the common speech impairments seen in HD include:

**Dysarthria:** This refers to a difficulty in producing clear and intelligible speech and is often caused by muscle weakness or difficulty with coordination and control [45]. Patients with HD may have difficulty forming words, and may have a slurred or slushy quality to their speech.
**Aphasia:** This refers to a difficulty with language and may include problems with understanding, producing, and comprehending speech. Patients with HD may have difficulty with word-finding, naming objects, or understanding complex sentences [46].
**Apraxia:** This refers to a difficulty with the planning and execution of voluntary movements, including speech movements [47]. Patients with HD may have difficulty with the coordination and sequencing of speech sounds, and may have difficulty producing certain sounds or words.

### 3.5. Biomarkers

Biomarkers are quantifiable markers of a particular biological process or condition, and they can be used to diagnose and monitor diseases [48,49]. In the context of HD, biomarkers can be used to provide additional information about the patient’s disease status, prognosis, and response to treatment. Some of the potential biomarkers of HD include:

**Neuropathological biomarkers:** Neuropathological biomarkers in HD encompass structural and molecular indicators of neurodegeneration. These include characteristic brain atrophy patterns, particularly in the basal ganglia and cortex, observed through neuroimaging techniques such as MRI [50]. Additionally, post-mortem examinations reveal protein aggregates, such as mutant huntingtin, in affected brain regions. Changes in neurotransmitter levels and neuronal connectivity also serve as biomarkers. These pathological features collectively offer a crucial understanding of the fundamental workings of HD, aiding in diagnosis, prognosis, and the development of targeted therapeutic strategies [51].

**Imaging biomarkers:** Imaging biomarkers in HD refer to quantifiable features observed through neuroimaging techniques such as MRI, PET, and CT scans [52]. These include structural indicators such as regional brain atrophy, particularly in the basal ganglia, cortex, and white matter tracts. Functional biomarkers reveal alterations in brain metabolism, connectivity, and neurotransmitter systems. Additionally, advanced imaging methods can detect microstructural changes in the brain’s white matter. These imaging biomarkers provide crucial insights into the progression and severity of HD, aiding in early diagnosis, tracking disease progression, and assessing the effectiveness of therapeutic interventions [53].

**Wet biomarkers:** Wet biomarkers in HD pertain to biological substances such as blood, cerebrospinal fluid (CSF), or tissue samples that are analyzed in a laboratory setting. These biomarkers offer insights into the biochemical and molecular changes associated with the disease [54]. In HD, wet biomarkers may include measurements of specific proteins or genetic material indicative of disease progression. For instance, elevated levels of mutant huntingtin protein fragments or alterations in certain neurotransmitters can serve as wet biomarkers. Additionally, RNA or DNA analysis from biological samples can provide genetic information relevant to HD diagnosis and progression. These wet biomarkers hold promise for improving the early detection and monitoring of HD [53].

#### 3.5.1. Brain Imaging

Brain imaging plays a crucial role in understanding and diagnosing HD. Several imaging techniques offer valuable insights into the structural and functional alterations that take place in the brains of individuals affected by this neurodegenerative disorder [55]. One of the primary imaging modalities used in HD is magnetic resonance imaging (MRI). Structural MRI scans unveil important information about the brain’s anatomy, allowing clinicians to detect specific abnormalities associated with HD [56]. Common findings on MRI include atrophy of the striatum, a region deep within the brain that is significantly affected by the disease. This atrophy is particularly pronounced in the caudate nucleus and putamen, which play a role in regulating physical movement and coordination. As the disease progresses, these regions shrink, and the ventricles (fluid-filled spaces in the brain) may enlarge. These structural changes are visible on MRI and contribute to the diagnosis of HD.

Imaging methods that assess functionality, such as positron emission tomography (PET) and functional MRI (fMRI), provide insights into the brain’s activity and connectivity. PET scans can reveal metabolic changes in the brain, including reduced glucose metabolism in affected areas. This reduction in metabolic activity corresponds to the regions of atrophy seen on structural MRI scans and is indicative of the dysfunction occurring in those brain areas. Functional MRI, on the other hand, can assess the connectivity and interaction between different brain regions [57]. In HD, disruptions in functional linkage between the basal ganglia as well as other areas of the brain contribute to the motor and cognitive symptoms of the disease. These functional imaging techniques not only aid in diagnosis but also provide valuable information for tracking disease progression and evaluating potential treatments. Furthermore, progress in neuroimaging has resulted in the creation of specialized techniques for studying specific aspects of Huntington’s disease. Diffusion tensor imaging (DTI) is employed to evaluate the integrity of bundles of white matter fibers in the brain, which are essential for transmitting signals between different brain regions [58]. In HD, DTI can detect abnormalities in white matter connectivity, contributing to the understanding of how the disease affects information transfer within the brain. Additionally, some studies have employed functional connectivity MRI (fcMRI) to explore changes in brain networks and their role in HD symptomatology.

#### 3.5.2. EEG signal

Electroencephalography (EEG) is a valuable neurophysiological tool used in the diagnosis and assessment of HD. HD is a progressive neurodegenerative disorder that primarily affects the basal ganglia and results in widespread brain dysfunction [59]. While the determination of HD is primarily using clinical standards as a foundation, EEG has emerged as a complementary diagnostic tool to provide insights into the brain’s electrical activity and to detect certain characteristic patterns associated with the disease.

The proportionate strength of the narrow sub-band within the theta-alpha range (specifically 7–8 Hz) showed a statistically significant reduction in pre-HD subjects when compared to normal controls. This reduction in relative power of the 7–8 Hz sub-band suggests abnormal electrical activity in the brain of pre-HD individuals [60]. This slowing of brainwave activity is indicative of widespread cortical dysfunction and is often associated with cognitive impairment, which is a common feature of HD. Additionally, HD patients may exhibit increased interhemispheric asymmetry in their EEG patterns, reflecting the disruption of communication between the brain’s hemispheres [61]. These anomalies in EEG patterns are detectable during the early stages of the disease, occurring prior to the emergence of obvious motor symptoms, making EEG a potentially valuable tool for the purpose of early detection and monitoring the advancement of the condition.

Moreover, EEG can be particularly useful in differentiating HD from other neurodegenerative disorders with similar clinical presentations, such as Parkinson’s disease. HD often presents with chorea, a type of abnormal involuntary movement, which can be challenging to differentiate from Parkinson’s disease-related tremors [62]. EEG can aid in this differentiation by showing characteristic differences in the patterns of electrical brain activity between these disorders. While EEG findings in HD are not specific to the disease, the combination of clinical symptoms and EEG abnormalities can contribute to a more accurate diagnosis and help clinicians rule out other conditions. Furthermore, EEG may have a role in tracking advancement of the condition and evaluating the impacts of potential treatments for HD, providing valuable insights into the neurological changes associated with this devastating disorder.

### 3.6. Sleep Disorders Present in Huntington’s Disease

Sleep disturbances are common and often overlooked aspects of HD, a neurodegenerative disorder [63]. Individuals with HD frequently experience a range of sleep disorders that further complicate their already challenging condition. Insomnia is one of the most prevalent sleep issues in HD, marked by trouble initiating sleep or staying asleep. The motor symptoms of HD, including chorea and dystonia, can contribute to nighttime restlessness, making it challenging for affected individuals to achieve a restful night’s sleep. This chronic sleep disruption can exacerbate other symptoms of HD, such as cognitive and psychiatric disturbances, ultimately impacting the overall quality of life.

Another prominent sleep disorder in HD is excessive daytime sleepiness. This excessive drowsiness can be ascribed to a combination of various elements, encompassing disrupted sleep patterns at night and the disease’s impact on the brain’s structures involved in regulating wakefulness. Excessive daytime sleepiness not only impairs daily functioning but can also lead to an increased risk of accidents and falls. Moreover, individuals with HD may experience irregularities in their biological clocks, resulting in irregular sleep–wake cycles. These disturbances can contribute to daytime sleepiness and further affect their ability to engage in daily activities and maintain social connections. Furthermore, sleep disorders in HD can manifest as parasomnias, which involve abnormal behaviors or movements during sleep. Parasomnias in HD may include restless leg syndrome, periodic limb movements, or even sleepwalking. These behaviors can result in injury or disrupt the sleep of bed partners or family members. Additionally, sleep disorders in HD can exacerbate cognitive decline and behavioral disturbances, making it essential for healthcare professionals to address sleep issues as part of the comprehensive care plan for individuals with HD. Managing sleep disorders in HD often requires a multi-faceted approach, including medication, behavioral interventions, and lifestyle adjustments, to help improve the overall quality of sleep and mitigate their impact on the disease’s progression.

#### 3.6.1. Rapid Eye Movement Sleep Behaviour Disorder

This is a prevalent sleep disturbance linked with Huntington’s disease [64]. Rapid Eye Movement Behavior Disorder (RBD) is a sleep condition characterized by the absence of typical muscle paralysis during the REM (Rapid Eye Movement) phase of sleep. In individuals with RBD, the muscles of the body are not paralyzed during REM sleep, which allows individuals to act out their dreams. This can lead to sleep-related injuries and disrupted sleep [62]. The occurrence of RBD is more common in individuals with HD than in the general population, with estimates ranging from 30–50%. The development of RBD in HD is thought to be related to the underlying neurodegeneration that occurs in the disease. The loss of neurons in specific brain regions, such as the basal ganglia, may lead to the development of RBD in HD. The symptoms of RBD in HD can include violent movements during sleep, sleep-related injuries, and disrupted sleep [65]. The presence of RBD in HD can have a substantial influence on the overall well-being of individuals with the disease and can contribute to the development of additional sleep conditions, such as sleep-related breathing disorders.

**Characterization:**

REM sleep in individuals with HD exhibits distinct characteristics that set it apart from normal sleep patterns. Here are some key features:

- Loss of Atonia: In HD, there is a notable absence of muscular activity typically observed during the rapid eye movement (REM) sleep phase. This means that the characteristic muscle paralysis that occurs during REM, preventing individuals from acting out their dreams, is compromised. This leads to vivid and often vigorous movements or behaviors during this phase of sleep.
- Complex Motor Behaviors: Individuals with HD may exhibit a range of complex motor behaviors during REM sleep. These can include purposeful movements, such as reaching, grasping, or even more dramatic actions such as punching or kicking. These behaviors can be disruptive and may lead harm to the individual or their sleeping companion.
- Frequency and Intensity: The frequency and intensity of REM sleep behavior disorder (RBD) in HD can vary among individuals. Some may experience sporadic episodes, while others may have more frequent and intense behaviors. Factors such as the stage of HD progression and individual differences in sleep patterns may contribute to this variation.
- Dream Enactment: RBD in HD often involves vivid and often violent dream enactment behaviors. These behaviors are typically related to the content of the dream, suggesting a failure in the normal inhibitory mechanisms that prevent motor activity during REM sleep.
- Potential Prodromal Sign: Emerging research suggests that RBD might occur before the appearance of motor symptoms in Huntington’s disease. This has led to speculation that RBD could serve as a potential prodromal sign or early marker of the disease. Monitoring REM sleep behaviors in individuals at risk for HD could provide valuable insights into disease progression.

#### 3.6.2. Restless Legs Syndrome and Periodic Limb Movement

Restless legs syndrome (RLS) and periodic limb movement disorder (PLMD) are two separate sleep disorders that can affect people with Huntington’s disease [66]. This neurological condition is marked by an uncontrollable need to move the legs, frequently accompanied by uncomfortable sensations in the lower limbs [67]. It typically occurs during periods of inactivity and can interfere with sleep, leading to daytime fatigue and other problems. RLS is thought to be caused by abnormal brain activity involving the neurotransmitter dopamine. PLMD, on the other hand, is a sleep disorder characterized by repetitive, unintentional leg motions (and sometimes the arms) during sleep. These movements can cause disrupted sleep and can have connections to other sleep-related conditions such as sleep apnea. While the precise origin of PLMD remains unclear, it is thought to be related to abnormal brain signaling involving the neurotransmitter acetylcholine. Both RLS and PLMD can be treated with medications, such as dopamine agonists or benzodiazepines, to help reduce the frequency and severity of symptoms.

**Characterization:**

- Unpleasant Sensations: Individuals may experience uncomfortable sensations in their legs, described as tingling, crawling, or aching. These sensations are often accompanied by an uncontrollable desire to shift or reposition the legs or other limbs, especially when at rest.
- Worsening in the Evening/Night: Symptoms tend to worsen in the evening and at night, disrupting the ability to fall asleep and maintain restful sleep.
- Motor Restlessness: This includes both voluntary and involuntary movements of the lower limbs, which can extend to other parts of the body. These movements can be rhythmic and repetitive in nature.
- Periodic Movements: In addition to the continuous urge to move, individuals may experience periodic, involuntary limb movements during sleep. These movements often happen at consistent time intervals, typically occurring approximately every 20 to 40 s.
- Impact on Sleep Quality: Both RLS and PLMD can severely disrupt sleep, leading to insomnia, daytime fatigue, and impaired cognitive functioning.
- Bed Partner Awareness: In the case of PLMD, the affected individual is often unaware of the limb movements during sleep. It is usually a bed partner or a sleep study that observes these movements.
- Daytime Consequences: Both conditions can lead to daytime sleepiness and decreased the standard of life resulting from disrupted sleep architecture.

**Automatic Detection:**

The diagnosis of RLS and PLM is based on patient self-reporting, and polysomnography (PSG), which is an overnight sleep study that gathers a range of physiological metrics, such as brainwave patterns, ocular movements, and muscle responses. PSG is the gold standard for diagnosing RLS and PLM, but it is expensive, time-consuming, and requires specialized equipment and trained technicians . Over the past few years, there has been a noticeable trend in growing the interests to develop automated methods for detecting RLS and PLM using non-invasive sensors, such as accelerometers, which can be worn on the leg or ankle. These sensors can detect movement and provide objective measures of leg movements during sleep [68].

One approach to automatic detection of RLS and PLM is to use AI-powered techniques, which can be trained to recognize patterns in the accelerometer data that are associated with RLS and PLM. This approach has shown promising results in several studies, with accuracy rates ranging from 80% to 90%. Another approach to automatic detection of RLS and PLM is to use signal processing techniques to analyze the accelerometer data [69]. This approach involves extracting various features from the accelerometer data, such as the frequency and amplitude of the leg movements, and using these features to identify RLS and PLM. This approach has also shown promising results in several studies, with accuracy rates ranging from 70% to 90%.

## 4. Exploring HD Disease Diagnosis via AI-Powered Models

### 4.1. Preamble—Diagnosis of Huntington’s Disease via AI-Powered Models

The need for AI-powered approaches in HD diagnosis arises from the pressing demand for early and accurate detection of this debilitating neurodegenerative disorder. HD is a multifaceted condition with a broad range of clinical manifestations, making it challenging for clinicians to diagnose, particularly in its early stages. ML and DL models have demonstrated their potential in handling the intricate and multi-modal data associated with HD, including genetic information, neuroimaging scans, and clinical assessments. These models can leverage vast datasets to identify subtle patterns and biomarkers that might elude human observers, enabling earlier and more precise diagnosis. Additionally, the development of automated diagnostic tools can alleviate the burden on healthcare professionals, streamline the diagnostic process, and ultimately lead to better patient outcomes. Given the ongoing progression of HD coupled with the lack of a definitive cure, the timely diagnosis facilitated by ML and DL techniques becomes paramount for initiating appropriate interventions, providing counseling, and advancing research into potential therapies. Therefore, the integration of ML and DL models into HD diagnosis is not only a necessity but also holds significant promise for enhancing the quality of life for individuals and families impacted by this devastating disease.

### 4.2. Machine Learning Techniques

Machine learning techniques have emerged as valuable tools in the diagnosis and assessment of Huntington’s disease. These techniques utilize various algorithms and computational approaches to analyze complex data sets, offering clinicians and researchers new insights into the disease [70]. One of the primary applications of machine learning in HD diagnosis is the identification of biomarkers and patterns within medical images, such as magnetic resonance imaging (MRI) and functional MRI (fMRI) [71]. Machine learning algorithms can detect subtle changes in brain structure and function, helping to distinguish individuals with HD from healthy controls and providing a means to monitor disease progression over time. Additionally, machine learning models can analyze clinical data, including motor, cognitive, and psychiatric assessments, to identify relevant features and patterns that contribute to accurate diagnosis and prognosis [72].

It also plays a crucial role in predictive modeling for HD risk assessment. By incorporating genetic data and other relevant factors, machine learning algorithms can predict an individual’s likelihood of developing HD, aiding in early intervention and counseling. Furthermore, machine learning approaches can be applied to large-scale genetic studies to identify genetic modifiers and factors linked to the potential for risk involving with the age of onset and disease advancement. This information not only deepens our understanding of the disease but also has implications for the development of targeted therapies. In summary, machine learning techniques are advancing the field of HD diagnosis by facilitating the extraction of valuable insights from clinical and genetic data, ultimately leading to earlier detection and improved management of this devastating neurodegenerative disorder.

#### 4.2.1. Naive Bayes

The Naive Bayes classifier stands out as a prominent choice for effectively discerning gait signals between individuals with HD and those without the condition [73]. The study reports an impressive accuracy rate of 94.4% achieved by the Naive Bayes classifier in this diagnostic context. This highlights the effectiveness of Naive Bayes as a valuable machine learning tool for Huntington’s disease diagnosis, offering the potential for non-invasive and objective assessment of individuals based on their gait dynamics, aiding in the timely identification and continuous tracking of the condition.

#### 4.2.2. Decision Tree

The use of Decision Tree stands out as a highly effective tool in the diagnosis of Huntington’s disease [73]. Decision Tree achieved an impressive average accuracy of 100% in accurately classifying gait signals from subjects with HD. This remarkable accuracy underscores the robustness of the Decision Tree algorithm in distinguishing individuals with HD based on their gait dynamics. Additionally, the Decision Tree emerges as a pivotal machine learning algorithm employed for the prediction and identification of potential contributing genes in Huntington’s disease [74]. This method involves the use of Decision Tree to formulate rules for attributes, specifically genes, and makes determinations regarding the prediction class, which denotes whether a given sample is associated with HD or not. Remarkably, the Decision Tree model showcased its efficacy by achieving an impressive cross-validated classification accuracy of 90.79% with a standard deviation of 4.57% when applied to the expression data of prefrontal cortex samples.

#### 4.2.3. Support Vector Machine

Support Vector Machine (SVM) emerges as a crucial classifier for gait classification, playing a significant role in the context of Huntington’s disease diagnosis, alongside other pathological conditions [75]. The utilization of SVM to differentiate gait patterns among diverse clinical groups, including individuals with Huntington’s disease, post-stroke patients, and healthy elderly individuals, employing data collected from inertial sensors. The classifier is trained using features derived from subject-specific Hidden Markov Models (HMMs), encompassing temporal and frequency domain signal data, and employs a leave-one-subject-out cross-validation technique, working in conjunction with three HMMs to assess likelihoods and ensure precise gait classification. The SVM also emerges as a valuable supervised classification method utilized for the discrimination of neurodegenerative diseases, including Huntington’s disease, through gait analysis [76]. This approach leverages SVM as a prediction model to classify and monitor these target diseases effectively. Notably, the SVM model aids in the identification of the most predictive features extracted from the gait analysis dataset, enabling a refined and accurate disease discrimination process. Impressively, the SVM model achieves a commendable accuracy rate of 86.9% in distinguishing these neurodegenerative diseases, underscoring its significance as a powerful tool for enhancing the diagnostic capabilities and understanding of HD and similar conditions through the analysis of gait patterns.

SVM is harnessed for classification purposes, specifically to distinguish individuals as either pre-HD or controls based on neuroimaging data [77]. Collaborating with linear discriminant analysis (LDA), SVM plays a crucial role in developing classification models capable of decoding essential information about the disease state from neuroimaging data. Impressively, these classification models utilizing SVM achieve notable success, reaching up to 76% accuracy in effectively distinguishing between pre-HD and control individuals based on their neuroimaging profiles. SVM is also applied for the classification of subjects by their HD stage, based on oculomotor features [78]. The accuracy of the SVM classifier varies depending on the specific classification task, achieving 73.47% accuracy for distinguishing control participants from pre-HD participants, 81.84% for distinguishing control participants from HD subjects, and 83.54% for distinguishing pre-HD subjects from HD patients, highlighting its effectiveness in stratifying individuals based on disease progression. Linear SVM is employed to classify eye tracking data across pre-HD, HD, and control groups, utilizing different combinations of features to optimize performance. Notably, the study reports the best accuracy of 76.88% for the CTRL vs. HD classifier and 72.50% for the pre-HD vs. HD classifier, underscoring the utility of SVM in Huntington’s disease diagnosis by leveraging oculomotor performance-derived features to accurately differentiate disease stages.

SVM plays a pivotal role in the classification of HD stages based on features extracted from T1- and diffusion-weighted imaging data [79]. Utilizing SVM, different feature selection techniques, such as whole-brain GM or FA values, subcortical regions-of-interest GM or FA values, and automated GM or FA value selection via the Relief-F algorithm, are employed to classify HD stages. This research showcases the adaptability of SVM, achieving noteworthy distinctions between Early-HD and Pre-HD or healthy individuals, with accuracy levels spanning from 85% to 95%. Moreover, SVM effectively discriminates Pre-HD from controls using the caudate region’s FA feature, achieving an accuracy of 74%.

SVM model is harnessed to facilitate the development of a novel formula for HD. SVM, a versatile machine learning algorithm widely recognized for its proficiency in classification and regression tasks, proves instrumental in handling the complex task of formulating a treatment for Huntington’s disease [80]. This research utilizes SVM to model training using documented Traditional Chinese Medicine (TCM) prescriptions. The objective is to identify a formula that can effectively target multiple proteins associated with HD, leveraging the SVM model’s ability to work with high-dimensional data and complex datasets. SVM is also utilized to classify gait signals from unknown subjects, distinguishing between those suffering from HD and healthy subjects [73]. Remarkably, experimental results highlight SVM’s outstanding performance, achieving an impressive average accuracy of 100.0% in accurately classifying gait signals. The remarkable precision achieved marks a notable milestone in Huntington’s disease diagnosis, underscoring SVM’s potency in effectively utilizing gait dynamics data for dependable differentiation of HD patients from non-afflicted individuals.

#### 4.2.4. Random Forest

In the ref. [74], Random Forest emerges as a prominent machine learning algorithm employed for the identification of contributing genes in Huntington’s disease. This strategy employs Random Forest to scrutinize postmortem prefrontal cortex samples from HD patients and control subjects, with the objective of identifying genes potentially linked to HD pathogenesis. The versatility of Random Forest proves beneficial in this context by effectively reducing the dimensionality of the data and highlighting the most relevant genes implicated in the pathophysiology of HD.The Random Forest model achieved a notable accuracy of 90.45 ± 4.24%, highlighting its pivotal role in aiding the diagnosis and comprehension of HD by deciphering the genetic components influencing its onset and progression. Random Forest also emerges as a prominent supervised classification model utilized for the discrimination of neurodegenerative diseases, including Huntington’s disease, through gait analysis. Random Forest serves as a computational classification technique, effectively characterizing these diseases using extracted features from gait cycles [76]. The study reports an impressive accuracy rate of 84.9% achieved by the Random Forest model, highlighting its substantial contribution to the accurate discrimination of HD and other neurodegenerative conditions based on gait patterns. The ref. [81] mainly focuses on assessing the significance and order of importance of potential factors that could predict the progression of clinical symptoms in patients with manifest HD. It accomplishes this by employing a random forest regression model to forecast how clinical outcomes change based on these factors.

Random Forest emerges as a powerful machine learning technique employed to discern microRNA biomarkers indicative of susceptibility to Juvenile Onset Huntington’s Disease (JOHD) [82]. The research employs the Random Forest methodology strategically to build predictive models, which can distinguish between JOHD and WT samples using mouse cortex samples from both young and aged groups. Additionally, it aims to forecast the inclination toward those genotypes. Impressively, the Random Forest model yields several robust models with testing accuracies exceeding 80% and impressive Area Under the Curve (AUC) scores surpassing 90%. It demonstrates a remarkable ability to distinguish between JOHD and WT samples, featuring a mature mRNA-based model that achieves a flawless 100% AUC score, highlighting its outstanding discriminatory capabilities. This application of Random Forest in the study underscores its potential in not only identifying crucial microRNA biomarkers but also in the diagnosis and predisposition assessment of Juvenile Onset HD, offering a significant advancement in the field of HD diagnostics.

#### 4.2.5. K-Nearest Neighbours

K-Nearest Neighbors (KNN) emerges as a significant classifier for the diagnosis of HD based on gait dynamics information. KNN, a well-known machine learning classifier, plays a crucial role in distinguishing individuals with HD from healthy subjects by classifying gait signals from unknown subjects [73]. The study reports an impressive accuracy rate of 97.2% achieved by the KNN classifier, underscoring its effectiveness in accurately identifying and differentiating individuals with HD from those without the condition through the analysis of gait dynamics.

KNN is employed for the identification of HD through audio signal processing [83]. KNN is applied in the classification stage following dimensionality reduction of voice signals, contributing to the accuracy of disease detection. The study demonstrates the effectiveness of the combination of the emobase2010 feature extractor with the KNN classifier, achieving an impressive accuracy rate of 97.3%. Notably, this high accuracy is achieved while maintaining a prediction time below one second, highlighting the practical utility of KNN in Huntington’s disease diagnosis through audio signal analysis.

#### 4.2.6. Ensemble Models

The ref. [84] highlights the crucial significance of ensemble classifier algorithms, with a specific emphasis on employing general ensemble classifier algorithms, in distinguishing gait patterns between individuals affected by HD and those who are healthy. This innovative methodology amalgamates individual classifier algorithms such as Logitboost and RandomForest, where Logitboost serves as the metaclassifier and RandomForest acts as the base classifier. The combination of Logitboost and RandomForest as ensemble classifiers showcases superior performance, particularly outperforming other tree decision algorithms in effectively classifying HD gait data. Significantly, the ensemble classifier method introduced demonstrates notable improvements in accuracy. It successfully classified 13 out of 14 subjects correctly and accurately identified all seven individuals with HD when employing the Logitboost and RandomForest combination. This showcases the significant promise of ensemble classifiers, specifically in harnessing ankle-mounted iPhone sensor data for robust diagnostic capabilities within the domain of Huntington’s disease classification. This approach holds substantial promise for advancing the accuracy and efficiency of HD diagnosis through gait analysis.

This research [85] presents an ensemble machine learning model that consistently outperforms nine conventional machine learning models, notably excelling in terms of accuracy. This ensemble model achieves a commendable balanced accuracy of 55.3% ± 6.1 in a 4-group classification of HD progression states. Even more impressive results are observed in binary classifications, with accuracies ranging from 70.9% ± 9.4 to 83.3% ± 6.3. Notably, the accuracy of the ensemble model experiences further augmentation through the incorporation of volumetric scores from diverse brain regions, including the occipital cortex, lateral ventricles, cingulate, and temporal lobe, in addition to the striatal structures. This emphasizes the potential of ensemble learning algorithms in advancing the precision of HD diagnosis through the utilization of structural MRI data, illustrating a significant stride forward in the field of neuroimaging-based diagnostics.

#### 4.2.7. Automatic Machine Learning

AutoML provides a significant advancement by automating the selection and optimization of machine learning models, thus reducing the need for manual intervention in model selection and tuning [86]. Within this investigation, the utilization of auto-sklearn, which harnesses Bayesian optimization algorithms, effectively pinpoints the most proficient model within the training dataset. This optimization contributes to an elevated level of effectiveness and precision in prediction outcomes. Notably, the utilization of AutoML enables the integration of various speech features with demographic variables to predict cognitive, motor, and functional scores in HD. Moreover, it supports the creation of fully automated methods for speech analysis, potentially minimizing the need for manual annotations and enabling remote assessment of individual conditions in Huntington’s disease and similar neurodegenerative disorders. While the paper does not explicitly mention the accuracy of the AutoML models, it emphasizes the significant improvement in predictions when combining speech features with demographic variables, showcasing its potential for accurate assessment in HD. This innovative approach holds immense promise for advancing the diagnostic capabilities of Huntington’s disease.

#### 4.2.8. Summary of Machine Learning Models

In summary, machine learning models have emerged as powerful tools for the diagnosis and understanding of HD. Various ML algorithms, as described in the earlier headings, have been applied to diverse data sources such as gait dynamics, genetic information, neuroimaging data, and speech recordings to enhance HD diagnosis and prognosis as in Table 1. These models have shown remarkable accuracy rates, often surpassing 90%, and have the potential to contribute to early detection, monitoring, and understanding of HD. However, several limitations persist across these studies, including the need for larger and more diverse datasets, the interpretability of complex models, and ethical considerations related to data privacy and security. Moreover, generalization to larger populations and clinical settings remains a challenge. The incorporation of automatic machine learning (AutoML) approaches signifies a promising direction in automating model selection and parameter tuning, potentially making these ML models more accessible for clinical deployment. Overall, ML models offer substantial potential for improving HD diagnosis, but further research and validation are needed to fully harness their capabilities and ensure their clinical utility.

### 4.3. Deep Learning Techniques

Deep learning approaches have become potent instruments for advancing the field of HD research and diagnosis. These techniques utilize the artificial neural network (ANN) with stacked layers to automatically acquire complex patterns and representations from complex datasets. Deep learning models excel at capturing hierarchical and abstract features from diverse data sources, such as neuroimaging scans, genetic data, and clinical assessments. The application of deep learning in the context of HD has shown promise in enhancing diagnostic accuracy, predicting disease progression, and uncovering fresh perspectives on the fundamental mechanisms behind disorder.

#### 4.3.1. Artificial Neural Network

The study [87] introduces a mathematical model, incorporating Artificial Neural Networks (ANN), which effectively simulates HD disorders and accurately replicates the behavior of individuals affected by Huntington’s disease. Specifically, the ANN within the model is trained using comprehensive data and physiological insights concerning the Basal Ganglia (BG), the region of the brain primarily impacted by HD. This innovative model serves as a potent analytical tool for comprehensively studying HD behavior, offering valuable insights into the underlying causes of movement disorders in HD patients. By employing ANN in mathematical models of brain performance, particularly within the context of BG in HD, this research significantly contributes to the expansion of medical knowledge and sheds crucial illuminate the intricacies of brain function in individuals grappling with Huntington’s disease.

The research [88] introduces an innovative hybrid model designed to assess the symptoms of individuals afflicted with Huntington’s disease. This model ingeniously combines the robust predictive capabilities of an ANN with the interpretability afforded by a fuzzy logic system (FLS). Remarkably, the ANN component of the model achieved an impressive regression R value of 0.98, along with a low mean squared error (MSE) of 0.08. These metrics affirm the accuracy of the model in predicting the functional capacity level (FCL) of an individual. Complementarily, the FLS component offers a conclusive evaluation of the subject’s reaction condition, further enhancing the model’s interpretability. This amalgamation of ANN and FLS in the hybrid model enables a comprehensive evaluation of HD symptoms, effectively leveraging both predictive capabilities and linguistic interpretation. This pioneering model holds significant potential in advancing the daily lives of HD patients, offering a means to monitor and predict disease progression for improved care and management.

In the ref. [89], a comprehensive model employing a range of Artificial Neural Network (ANN) models to analyze data gathered from smart devices, such as smartphones or tablets, in order to forecast the functional capacity level of individuals afflicted with HD, is introduced. This approach encompasses a diverse array of ANN models, including Cascade forward backpropagation (CFBP), Feed-forward backpropagation (FFBP), Elman, Generalized regression neural network (GRNN), Nonlinear autoregressive exogenous model (NARX), Layer recurrent neural network (RNN), and Feed-forward time delay neural network (FFTDNN). The paper intricately details the entire process, from data preparation and labeling to the selection of learning algorithms, specific neural network training, performance evaluation, and comparative analysis. This study represents a significant stride toward leveraging advanced technology for a more precise and insightful assessment of functional capacity levels in HD patients.

The ref. [90] underscores the significance of employing non-linear techniques, particularly ANNs, as a potent tool in comprehending the intricacies of HD. The authors present a pioneering approach utilizing ANNs to accurately discern between control subjects and those affected by HD, leveraging DNA CpG methylation data. What sets this approach apart is its capacity to streamline the consideration of CpGs from hundreds of thousands down to a mere 237, showcasing the remarkable effectiveness of deep learning techniques in HD diagnosis. The study’s results unequivocally demonstrate that by focusing on these 237 CpGs and employing non-linear techniques such as ANNs, a precise differentiation between control and HD patients can be achieved. Overall, this paper advocates for the pivotal role of artificial neural networks, particularly as a deep learning technique, in the diagnosis of Huntington’s disease, particularly when leveraging DNA CpG methylation data.

#### 4.3.2. Deep Neural Network

Deep Neural Network revolutionizes the identification of HD through the utilization of DNN in analyzing speech signals [91]. The approach leverages a combination of acoustic and lexical features for automated detection. Employing a Leave-One-Subject-Out (LOSO) methodology, the DNN model is meticulously trained and validated, where individual subjects are consecutively held out as the test speakers. Notably, the study observes a progressive increase in the accuracy of this method, particularly with advancing disease stages. This underscores the potential of speech as an effective biomarker for monitoring HD progression. The performance evaluation of the DNN model, alongside other deep learning models, is quantified using the word error rate (WER), yielding an impressive range between 9.4 to 14.9. These results substantiate the notion that employing objective analyses through DNN and similar deep learning models holds significant promise in distinguishing between healthy individuals and those with HD. This advancement not only reinforces clinical diagnoses but also facilitates symptom tracking in non-laboratory and non-clinical settings, presenting a notable stride towards improved healthcare management for individuals affected by Huntington’s disease.

#### 4.3.3. Deep Convolutional Neural Network

The ref. [92] delves into the application of deep learning models, specifically VGG16 and 3D CNN, for diagnosing Huntington’s disease. The study reveals that VGG16, a well-established architecture, holds great promise in classifying disease severity through analyzing pressure data from individual footsteps, achieving an impressive 89% accuracy. Its proficiency in extracting nuanced features such as edges and corners significantly contributes to accurate classification. While VGG16 excelled, other techniques such as 3D CNN also demonstrated an accuracy of 82%. The study highlights that 3D CNN, though slightly less accurate at 82%, presents potential for improvement when combined with models such as VGG16. The paper suggests that while 3D CNN may have slightly different feature extraction capabilities compared to the novel model used, combining their strengths could lead to even more accurate Huntington’s disease diagnosis. This integrated approach signifies a promising stride towards refining disease classification, holding substantial implications for both clinical practice and research in this field.

#### 4.3.4. Extreme Learning Machine

Extreme Learning Machine (ELM) models, as outlined in this research, present a pioneering method for predicting the progression of Huntington’s disease based on brain scans. The approach, referred to as Brute-force Missing Data Extreme Learning Machine, showcases significant potential in this domain [93]. This novel method leverages the ELM framework to train models on datasets containing absent data for both processing and the desired outcomes. Notably, the ELM approach in this study demonstrates exceptional efficiency by individually constructing and training models for each sample in the test set. This process is remarkably efficient, eliminating the need for repeated access to the training data. Experimental comparisons conducted in the study reveal highly promising results, this underscores the effectiveness of employing ELM in the diagnosis of Huntington’s disease. By addressing missing data challenges and leveraging the power of ELM, this approach offers a significant stride forward in accurately predicting the progression of HD, holding considerable potential for advancing early diagnosis and intervention strategies for individuals affected by this condition.

#### 4.3.5. Deep Boltzmann Machine

The ref. [94] introduces a pioneering approach utilizing a stacked restricted Boltzmann machine (SRBM) in the analysis of RNA-seq data for Huntington’s disease diagnosis. This innovative deep learning technique is specifically tailored to identify key genes implicated in the progression of HD. By examining differentially activated neurons and changes in gene energy at various time intervals, SRBM efficiently screens disease-associated factors and genes. Experimental results underscore the remarkable efficacy of SRBM, demonstrating its ability to discern crucial information in time series gene expression datasets. This leads to a significant improvement in the accuracy of identifying disease-associated genes and predicting top-ranking genes, surpassing the capabilities of current state-of-the-art methods. Moreover, SRBM outperforms other computational approaches in analyzing gene expression data of HD-afflicted mice across distinct spans of time. Its automatic feature learning capacity, coupled with heightened precision in identifying disease-associated genes, underscores SRBM as a formidable tool in HD diagnosis. This approach stands at the forefront of computational methods, offering a highly effective means of understanding the genetic underpinnings of HD progression.

#### 4.3.6. Summary of Deep Learning Models

These algorithms represent a significant paradigm shift in the diagnosis and understanding of HD as in Table 2. These DL models excel in leveraging various data modalities such as gait dynamics, genetic information, neuroimaging data, speech signals, and RNA-seq data to enhance the accuracy and depth of HD diagnosis and prognosis. Notably, DL models such as DNNs showcase exceptional predictive capabilities, with impressive accuracy rates and the potential for monitoring HD progression. However, DL models often require large and diverse datasets for training, and their complexity may pose challenges in model interpretability and clinical applicability. Furthermore, while these models demonstrate remarkable potential, validation in diverse populations and clinical settings is essential to fully harness their capabilities and ensure their suitability for widespread clinical deployment. Nonetheless, DL models signify a transformative advancement in the field of HD diagnosis, offering valuable insights and paving the way for more accurate and personalized care for individuals affected by this debilitating disease.

## 5. Open Challenges

### 5.1. Setbacks in Computational Machine Learning Approaches

**Figure 4:**
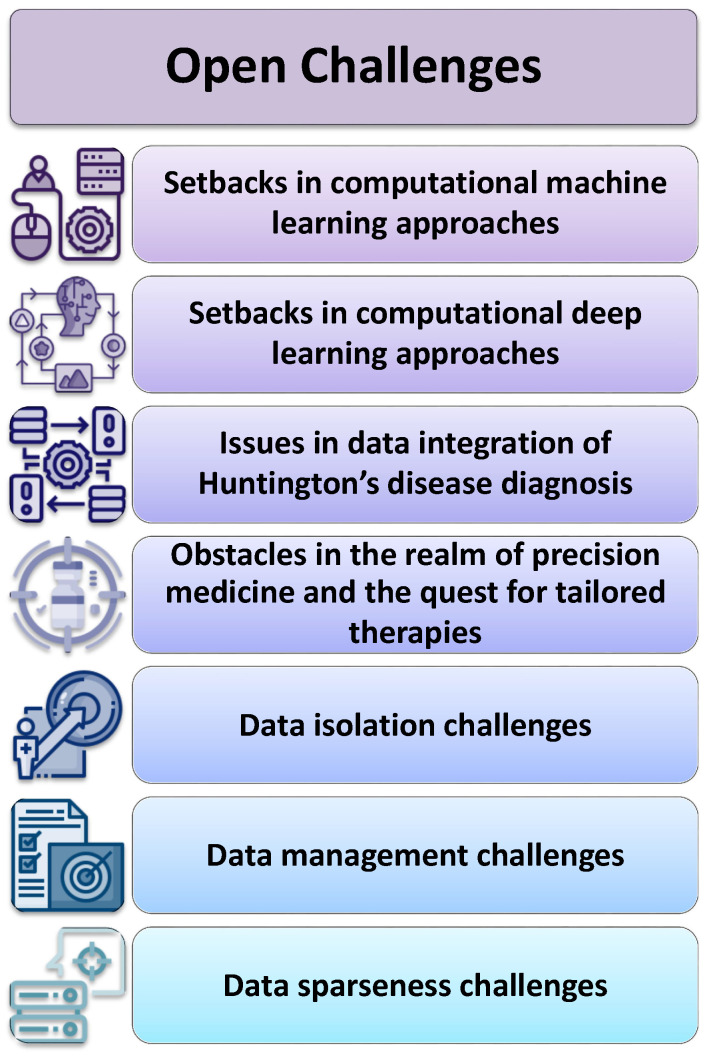
Open Challenges.

Computational machine learning (ML) models hold great promise for HD diagnosis, but they also face several significant challenges as shown in Figure 4 and it has been addressed for effective implementation and clinical utility:

**Data Availability and Quality:** One of the primary challenges in developing ML models for HD diagnosis is the availability of high-quality data. Gathering comprehensive datasets that include genetic, clinical, and imaging data from a diverse range of HD patients is essential. Ensuring the accuracy and completeness of these data is crucial for model training and validation.
**Data Imbalance:** HD is a rare genetic disorder and datasets for rare diseases that often suffer from class imbalance, with a limited number of positive cases (HD patients) compared to negative cases (healthy individuals). Datasets with imbalances can result in model bias, where the algorithm demonstrates strong performance in the majority class but struggles when dealing with the minority class. Addressing this imbalance is critical for accurate diagnosis.
**Feature Selection:** HD is a complex disorder with multifaceted clinical manifestations. Selecting the most informative features from various data sources (genetic, clinical, imaging) and determining their relevance to the disease diagnosis is a challenge. ML models need to incorporate relevant features while reducing dimensionality and noise.
**Interpretable Models:** Numerous machine learning algorithms, including deep learning models, are often referred to as “opaque” models, posing difficulties in deciphering the rationale behind their decisions. In the medical field, interpretability is crucial for understanding why a particular diagnosis or prediction was made. Developing interpretable models that provide insights into the disease process is essential.
**Ethical Concerns and Privacy:** Handling patient data in healthcare applications raises ethical and privacy concerns. Ensuring the security and privacy of sensitive health information while allowing for meaningful analysis is a delicate balance that must be addressed when developing ML models for HD diagnosis. Techniques such as federated learning, as highlighted in the work [95], have emerged as crucial tools in preserving privacy while enabling the collaborative analysis of medical data.
**Generalization and Validation:** ML models must generalize well to new, unseen data. Proper cross-validation techniques and external validation on diverse datasets are essential to ensure that the models’ performance remains consistent across different populations and settings.
**Early Detection and Biomarker Discovery:** Identifying reliable biomarkers for early HD detection is a significant challenge. ML models can assist in this process, but the discovery of biomarkers that can accurately predict disease onset or progression is an ongoing research area.
**Clinical Integration:** Transitioning ML models from research settings to clinical practice requires collaboration with healthcare professionals and regulatory bodies. Models need to be validated in clinical trials and integrated into existing healthcare systems, which can be a lengthy and complex process.
**Longitudinal Data:** HD is a progressive disease that evolves over time. Obtaining and analyzing longitudinal data is essential for tracking disease progression and treatment response accurately. ML models must be capable of handling longitudinal data effectively.
**Validation with Small Cohorts:** Due to the rarity of HD, it can be challenging to validate ML models with large cohorts of patients. Small sample sizes can lead to overfitting and may not capture the full spectrum of disease variability.

### 5.2. Setbacks in Computational Deep Learning Approaches

Deep learning (DL) models offer remarkable potential for advancing Huntington’s disease diagnosis and prognosis, yet they encounter several formidable challenges that demand careful consideration to realize their full clinical potential:

**Limited HD-Specific Data:** While data availability is a general challenge, obtaining a sufficiently large and diverse dataset specifically for HD is particularly challenging due to the rarity of the disease. HD-specific data, including genetic profiles, clinical records, and imaging data, may be limited in comparison to other more common medical conditions.
**Complex Disease Progression:** HD is a neurodegenerative disease with a highly complex and nonlinear progression pattern. Capturing this complexity in DL models, especially for early-stage diagnosis and monitoring, is challenging. DL models must account for the multi-modal nature of HD progression, including motor, cognitive, and psychiatric symptoms.
**Feature Extraction:** DL models often rely on automatic feature extraction from raw data. However, extracting meaningful features from complex data sources such as brain imaging (e.g., MRI, fMRI) and genetic data can be challenging. Developing effective feature extraction methods tailored to HD-specific data is essential.
**Heterogeneity of HD:** HD exhibits significant variability in symptom onset, progression, and severity among individuals. DL models need to account for this heterogeneity and provide personalized predictions and treatment recommendations, which can be challenging in clinical practice.
**Integration of Multiple Data Sources:** Effective HD diagnosis and prognosis may require integrating information from various sources, such as genetic, imaging, and clinical data. Developing DL models that can seamlessly integrate heterogeneous data and extract meaningful insights is a complex task.
**Model Explainability:** While interpretability is a general challenge in ML and DL, it is particularly critical in healthcare applications. DL models for HD diagnosis need to provide clear explanations for their predictions, helping clinicians to understand the rationale behind a diagnosis or prognosis.
**Scalability:** Training large DL models for healthcare applications often requires substantial computational resources. Ensuring that the models are scalable and can be deployed in resource-constrained clinical settings is a challenge.
**Clinical Adoption:** Even with accurate DL models, achieving widespread clinical adoption can be challenging. Overcoming barriers related to regulatory approvals, integration with electronic health records (EHRs), and gaining the trust of healthcare professionals is crucial for the successful deployment of DL-based diagnostic tools.
**Patient Data Privacy:** Protecting patient data privacy is a paramount concern. Ensuring that DL models comply with data protection regulations and securely handle sensitive patient information poses a challenge in healthcare applications.

### 5.3. Issues in Data Integration of Huntington’s Disease Diagnosis

The complexity of Huntington’s disease and the multitude of data sources involved pose a multifaceted challenge when it comes to the integration of diagnostic information. Firstly, there is a significant lack of standardized data formats and protocols across healthcare institutions, making it difficult to harmonize data from different sources. Additionally, the inherent privacy concerns surrounding patient information in healthcare pose a substantial hurdle, as ensuring data security and compliance with regulations such as HIPAA is paramount [96]. Furthermore, the heterogeneity of data types, ranging from clinical records and genetic profiles to neuroimaging data, demands sophisticated data integration techniques and tools to extract meaningful insights. The dynamic nature of the disease itself, with its variable progression and diverse symptomatology, complicates the process further, as the integration framework must be adaptable to changing data over time. Moreover, the scarcity of large-scale datasets for Huntington’s disease poses a challenge for training robust machine learning models, hampering the development of accurate diagnostic tools. Lastly, interdisciplinary collaboration between geneticists, clinicians, data scientists, and ethicists is essential to navigating these challenges successfully, emphasizing the importance of effective communication and teamwork in integrating HD diagnosis data for improved research and patient care.

### 5.4. Obstacles in the Realm of Precision Medicine and the Quest for Tailored Therapies

The pursuit of precision medicine and the identification of personalized treatments for HD confronts a multitude of formidable challenges [97]. Firstly, the inherent heterogeneity of the disease, with variations in onset age, symptom severity, and progression rates among individuals, makes it exceptionally complex to pinpoint universally effective treatments. The lack of comprehensive biomarkers that can accurately predict disease progression and treatment response further compounds this challenge. Additionally, the scarcity of large-scale, diverse patient datasets hinders the development of robust predictive models and therapies tailored to individual genetic profiles. Ethical concerns surrounding genetic testing and data privacy necessitate careful consideration, as patients and their families may be hesitant to share their genetic information for research purposes. The high cost and time-intensive nature of genomic sequencing and data analysis also limit the widespread adoption of precision medicine approaches. Collaborative efforts among researchers, clinicians, and pharmaceutical companies are imperative to surmount these challenges and unlock the potential of personalized treatments for HD, offering hope for improved patient outcomes and quality of life.

### 5.5. Data Isolation Challenges

Data isolation in HD diagnosis poses a significant impediment to holistic understanding and effective management of the condition. One primary challenge is the compartmentalization of patient data within various healthcare institutions, research centers, and databases. These silos of information inhibit the seamless sharing and integration of vital clinical, genetic, and neuroimaging data necessary for comprehensive diagnosis and treatment planning. Moreover, the lack of standardized data formats and protocols exacerbates this isolation, making it arduous to harmonize and compare data from different sources accurately. The inherent rarity of Huntington’s disease further compounds the problem, as it limits the pool of available patient data, hindering the development of robust diagnostic and predictive models. Privacy concerns surrounding patient information also contribute to data isolation, with stringent regulations and ethical considerations deterring the free exchange of genetic and clinical data. Overcoming these data isolation challenges in Huntington’s disease diagnosis necessitates collaborative efforts, the establishment of interoperable data-sharing frameworks, and the development of secure, privacy-preserving data-sharing solutions to facilitate more comprehensive and insightful analyses for improved patient care and research progress.

### 5.6. Data Management Challenges

The intricate landscape of Huntington’s disease diagnosis poses intricate data management obstacles, necessitating inventive solutions within the realms of clinical research and healthcare. One of the foremost challenges is the sheer volume and diversity of data involved. HD diagnosis requires the integration of various data streams, including clinical, genetic, and neuroimaging data. Managing this multidimensional data in a cohesive and standardized manner is crucial for accurate diagnosis and tracking of disease progression. Furthermore, the dynamic nature of HD necessitates the continuous acquisition and analysis of patient data over extended periods. This long-term data management involves maintaining data integrity, ensuring privacy and consent compliance, and adapting to evolving diagnostic criteria and treatment options. The need for secure and robust data storage and transmission systems becomes paramount, given the sensitive nature of genetic and clinical information. Interoperability and data sharing also pose significant challenges. Collaborative research efforts and multi-center clinical trials require seamless data exchange, but disparities in data formats and standards across institutions can hinder effective communication. Establishing data-sharing protocols while respecting patient privacy is a critical yet intricate task.

The incorporation of technologies such as machine learning and artificial intelligence into HD diagnosis introduces further complexities. Training algorithms and models for accurate prediction and early detection require large datasets, which may be scarce due to the rarity of HD cases. Ensuring the quality and representativeness of training data while addressing ethical considerations is a multifaceted challenge. The ethical implications of data management in HD diagnosis cannot be overlooked. Balancing the potential benefits of data sharing for research and treatment advancements with the need to protect patient privacy and confidentiality is an ongoing ethical dilemma. Addressing the data management challenges of HD diagnosis is pivotal in advancing our understanding of the disease, improving diagnostic accuracy, and developing effective treatments. Solutions must encompass data integration, security, interoperability, ethical considerations, and adaptation to evolving diagnostic paradigms to pave the way for better outcomes and hope for individuals and families affected by HD.

### 5.7. Data Sparseness Challenges

Diagnosing HD poses unique challenges, primarily due to the data sparseness associated with this devastating neurodegenerative disorder. HD is a rare genetic condition, affecting only a small portion of the population, which makes it challenging to accumulate a substantial dataset for research and diagnostic purposes [98]. The rarity of the disease means that there is limited access to clinical and genetic information, hindering efforts to develop accurate diagnostic tools and therapies. Moreover, HD is characterized by a long prodromal phase, during which subtle motor and cognitive symptoms emerge before a formal diagnosis can be made. This extended prodromal phase further complicates data collection, as individuals may not seek medical attention until their symptoms become more pronounced. Moreover, a considerable number of subjects who are at risk of Huntington’s disease may opt out of genetic testing because of the emotional and psychological toll that comes with knowing their genetic status. This decision contributes to the absence of data in certain cases. The genetic complexity of HD adds to the data sparseness challenge.

## 6. Future Potentials

**Figure 5:**
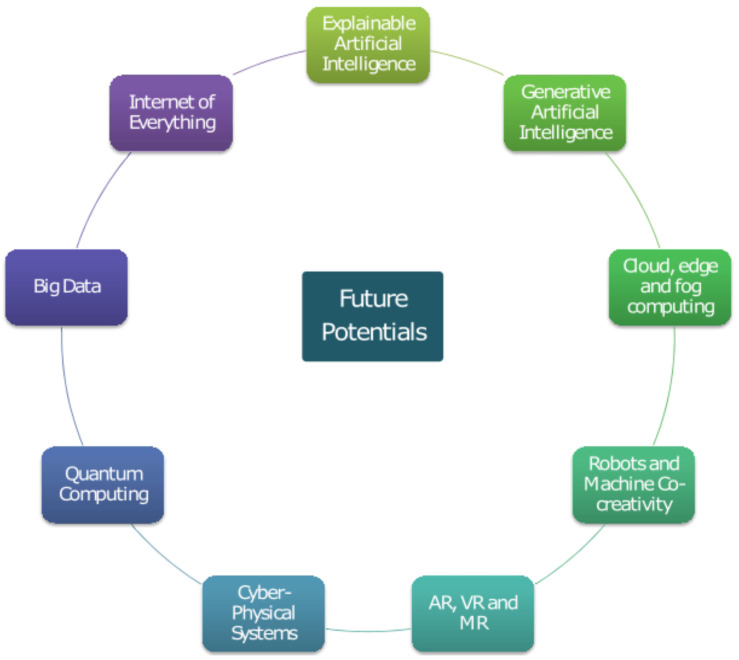
Future Potentials.

According to Figure 5, Huntington’s disease exhibits promising future potentials, hinting at evolving research pathways that offer prospects for improved management and therapeutic breakthroughs.

### 6.1. Explainable Artificial Intelligence

The application of Explainable AI (XAI) in Huntington’s disease diagnosis holds immense promise in enhancing our understanding of the disease’s complex diagnostic factors. XAI algorithms provide transparency and interpretability, crucial in unraveling the multifaceted nature of HD [99,100]. By leveraging machine learning techniques that can not only make accurate predictions but also provide comprehensible explanations for those predictions, XAI can help clinicians and researchers pinpoint the specific genetic markers, clinical features, and other factors contributing to an individual’s risk or progression of HD. This transparency not only aids in early and accurate diagnosis but also aids in the development of more personalized treatment plans. Moreover, XAI can facilitate the identification of subtle patterns in medical records and imaging data, aiding in the early detection of prodromal symptoms, thus potentially extending the window for intervention. Overall, the integration of Explainable AI in Huntington’s disease diagnosis not only enhances diagnostic accuracy but also advances our understanding of the disease’s underlying mechanisms, offering hope for improved management and eventually a cure for this devastating condition. Additionally, the work [101] on an Explainable Supervised Machine Learning Model for Predicting Respiratory Toxicity of Chemicals underscores the significance of explainable ML approaches in various biomedical domains, further emphasizing the critical role of interpretability in complex predictive models, as is applicable in the context of Huntington’s disease diagnosis.

### 6.2. Generative Artificial Intelligence

Emerging generative AI technologies, encompassing deep learning methods such as Generative Adversarial Networks (GANs), Vision Transformer (ViT), and Variational Autoencoders (VAEs), exhibit potential in diverse medical contexts, including their utility in diagnosing and predicting the course of diseases such as HD [102]. Huntington’s disease, stemming from a genetic mutation, is a neurodegenerative condition. Timely diagnosis plays a pivotal role in enhancing patient care and unlocking potential avenues for future therapeutic interventions [103]. Here is how generative AI can be used for HD diagnosis:

**Data Generation and Augmentation:** Generative AI can be used to generate synthetic medical images or data that closely resemble real patient data [104]. This could prove beneficial in training machine learning models in situations where actual patient data is scarce or in cases where privacy issues are a concern. Augmenting the dataset with generated data can improve the performance of diagnostic models.
**Image Enhancement:** Generative models can be used to enhance the quality of medical images, making it easier for healthcare professionals to identify subtle signs of HD in brain scans or other medical imaging data [105]. Enhanced images can provide better insights and aid in more accurate diagnoses.
**Early Detection:** Generative AI can assist in the early detection of HD by analyzing patterns in medical data over time. Longitudinal data from patients can be used to create generative models that predict the progression of the disease, allowing for early intervention and personalized treatment plans.
**Predictive Biomarkers:** Generative models can identify predictive biomarkers associated with Huntington’s disease. Through the examination of extensive patient data encompassing genetic profiles, medical records, and clinical evaluations, these models can unveil nuanced relationships that might remain concealed when employing conventional statistical methods.
**Disease Progression Modeling:** Generative AI can create models that simulate the progression of HD in virtual patients. This can be useful for understanding how the disease develops over time and for testing the efficacy of potential treatments in silico before clinical trials.
**Support for Clinicians:** Generative AI can assist clinicians in making more informed decisions by providing additional insights into patient data. For example, it can generate visualizations that highlight regions of interest in medical images or generate reports, summarizing key findings from patient records.
**Personalized Treatment Plans:** Leveraging data from a substantial group of individuals afflicted with HD, generative models have the potential to facilitate the development of personalized therapeutic strategies. These plans can take into account an individual’s genetic profile, disease progression, and response to previous treatments to optimize therapeutic strategies.
**Drug Discovery:** Generative AI can be applied to discover potential drug candidates for Huntington’s disease by generating molecular structures that may interact with specific disease-related targets. This can accelerate the drug development process.

### 6.3. Internet of Everything

The Internet of Everything (IoE) is a concept that extends the Internet of Things (IoT) by not only connecting devices and machines but also by incorporating data from people, processes, and data itself [106]. This interconnected network of physical objects, data, and people holds great potential for improving various aspects of healthcare, including the diagnosis and management of diseases such as HD. The use of IoE in HD diagnosis can have several significant benefits:

**Remote Monitoring:** IoE allows for continuous remote monitoring of patient’s vital signs and movements. Wearable devices, such as fitness trackers and smartwatches, can collect data on heart rate, sleep patterns, and physical activity [107]. In the case of HD, changes in motor function and sleep disturbances can be early indicators of disease progression. By continuously monitoring these metrics, healthcare providers can detect changes sooner and intervene proactively.
**Genomic Data Sharing:** The presence of a particular genetic mutation is responsible for HD, necessitating genetic testing as a common diagnostic procedure [108]. IoE can facilitate the sharing of genomic data securely between patients, clinicians, and researchers. Utilizing these data allows for the identification of individuals who may be susceptible to Huntington’s disease and enables the monitoring of the progression of the condition. Privacy and security measures must be robust to protect sensitive genetic information.
**Telemedicine and Consultations:** IoE enables telemedicine and remote consultations, making it easier for patients with HD to access specialized care. The utilization of video conferencing and remote monitoring can diminish the necessity for regular face-to-face appointments, especially benefiting individuals residing in distant or underserved regions.
**Data Analytics and Predictive Modeling:** IoE allows for the collection of vast amounts of patient data. Advanced data analytics and machine learning algorithms can process this information to identify patterns and trends associated with HD. Predictive modeling can help anticipate disease progression and develop personalized treatment plans.
**Medication Management:** For individuals with HD, managing medications is crucial. IoE can support medication adherence through smart pill dispensers and reminders [109]. These devices can notify patients when it is time to take their medication and send alerts to caregivers or healthcare providers if doses are missed.
**Support Communities:** IoE can connect HD patients with support communities and resources. Online forums, social networks, and communication tools can help patients and caregivers connect, share experiences, and access valuable information and emotional support.
**Clinical Trials and Research:** IoE can facilitate the recruitment and monitoring of participants in clinical trials for potential HD treatments. Real-time data collection can provide researchers with valuable insights into treatment efficacy and safety.

### 6.4. Big Data

The utilization of Big Data and Augmented Analytics has become instrumental in the detection and handling of intricate conditions such as Huntington’s disease. By tapping into extensive datasets encompassing patient data such as genetics, clinical histories, and even information from wearable devices, healthcare experts can uncover hidden patterns and connections that might go unnoticed otherwise, facilitating enhanced diagnosis and management [110]. By aggregating and analyzing this diverse dataset, machine learning algorithms can be trained to recognize subtle early-stage symptoms and risk factors associated with HD [111]. Augmented Analytics further enhances this process by providing user-friendly interfaces that enable healthcare providers, even those without extensive data science expertise, to explore and interpret the findings.

This combined approach enables earlier and more accurate diagnosis of HD, which is critical for timely intervention and patient care planning [112]. It also facilitates personalized treatment strategies by tailoring therapies based on an individual’s genetic profile and disease progression patterns. Additionally, the continuous monitoring of patients through wearable devices and real-time data analysis can provide insights into disease progression, enabling healthcare providers to make informed decisions about treatment adjustments. The synergy between Big Data and Augmented Analytics represents a promising avenue for improving the early diagnosis and management of Huntington’s disease, ultimately enhancing the quality of life for affected individuals and their families.

### 6.5. Cloud, Edge, and Fog Computing

Cloud, edge, and fog computing are all valuable technologies that can play significant roles in the diagnosis and management of diseases such as HD, which is a complex neurodegenerative disorder. These technologies offer unique advantages in terms of data processing, storage, and accessibility, allowing for more efficient and effective diagnostic and therapeutic approaches.

#### 6.5.1. Cloud Computing

**Data Storage and Centralization:** Cloud computing offers vast storage capabilities, making it ideal for storing large datasets such as genetic information, medical records, and imaging data related to Huntington’s disease patients [113]. Researchers and healthcare providers can securely store and access these data from anywhere with an internet connection.
**Data Analysis:** Cloud platforms offer the computational muscle essential for intricate data analysis, including genetic sequencing, medical imaging analysis, and machine learning algorithms for early HD diagnosis. Researchers can run resource-intensive computations on the cloud, accelerating the development of diagnostic tools and treatment options.
**Collaboration:** Cloud-based platforms facilitate collaboration among researchers and clinicians from different locations, allowing them to share data, insights, and best practices in diagnosing and managing HD. This collaborative approach can lead to faster advancements in HD research and treatment.

#### 6.5.2. Edge Computing

**Real-time Data Processing:** Edge computing brings computation closer to the data source, making it ideal for real-time analysis of patient data, including wearable device data, continuous monitoring, and sensor data [114]. In the context of HD, edge devices can process and analyze patient data on the spot, providing immediate feedback to both patients and healthcare providers.
**Data Privacy and Security:** The utilization of edge computing can fortify data privacy and security by maintaining sensitive patient data in proximity to its origin. This approach mitigates the potential for data breaches, preserving the confidentiality of patient information, a pivotal concern in healthcare environments.
**Reduced Latency:** For applications such as telemedicine or remote monitoring of HD patients, edge computing reduces data transmission latency, enabling quicker response times and ensuring that critical information reaches healthcare professionals promptly.

#### 6.5.3. Fog Computing

**Distributed Processing:** Fog computing extends the capabilities of edge computing by enabling distributed data processing and analysis [115]. In HD diagnosis, fog computing can distribute processing tasks across multiple edge devices, optimizing computational resources for complex tasks.
**Resilience:** Fog computing provides redundancy and resilience in data processing, ensuring that critical diagnostic processes continue to operate even in the event of a device failure [116]. This reliability is essential for continuous monitoring and early detection of HD symptoms.
**Scalability:** Fog computing possesses inherent scalability, readily expanding its capacity to cater to a rising population of HD patients and an expanding array of devices. With the proliferation of patients and data sources, fog computing exhibits the adaptability required to adeptly and effectively handle the augmented workload.

### 6.6. Robots and Machine Co-Creativity

By harnessing advanced robotics and artificial intelligence, healthcare professionals can collaborate with machines to enhance diagnostic capabilities. Robots equipped with sophisticated imaging technologies can perform highly detailed scans of the brain, capturing minute structural and functional changes that are indicative of Huntington’s disease [117]. These machines can work tirelessly, ensuring a comprehensive analysis of patient data. Moreover, AI-driven algorithms can sift through vast datasets, quickly identifying patterns and anomalies that may escape the human eye.

The true power of machine co-creativity lies in its ability to augment human expertise. By working in tandem with healthcare providers, these technologies can offer valuable insights and suggestions, helping clinicians make more accurate and timely diagnoses. This collaborative approach also enables a multidisciplinary team of experts to pool their knowledge and refine diagnostic criteria continuously. Furthermore, the integration of robotics and AI can streamline the diagnostic process, reducing the time and resources required for assessments. This is particularly crucial for HD, which currently lacks a cure but benefits significantly from early detection and management. Their ability to enhance accuracy, efficiency, and collaboration among healthcare professionals holds the promise of earlier interventions and improved patient outcomes in the battle against this devastating disorder.

### 6.7. Quantum Computing

The vast potential of quantum computing is poised to drive significant progress in the healthcare sector, offering promising prospects for improving the diagnosis and therapeutic interventions for a multitude of illnesses [118,119]. Quantum computing could be beneficial in this context:

**Efficient Genetic Analysis:** Quantum computers have the capability to process vast amounts of genetic data much faster than classical computers. This becomes particularly pertinent when dealing with HD, where the focus lies on scrutinizing the patient’s DNA to pinpoint the distinct genetic mutation responsible for this condition. Quantum computers can accelerate the process of genetic sequencing and analysis, potentially leading to quicker and more accurate diagnoses.
**Simulating Protein Structures:** Understanding the molecular basis of diseases such as Huntington’s relies on simulating complex protein structures. Quantum computers can simulate these structures with far greater precision and speed than classical computers. This potential can assist scientists in uncovering the underlying processes of the condition, potentially pinpointing areas for therapeutic intervention.
**Drug Discovery:** Quantum computing can expedite drug discovery by simulating the interactions between potential drug compounds and the target proteins involved in HD. This has the potential to substantially decrease the resources and time required for the development of novel therapies or the discovery of already-available drugs suitable for repurposing in treatment.
**Personalized Medicine:** Quantum computing can enhance the personalization of treatment plans. By analyzing a patient’s genetic data alongside other clinical parameters, quantum computers can help healthcare providers tailor treatment strategies specifically to each patient’s unique genetic makeup, potentially leading to more effective treatments and better outcomes.
**Data Security:** As quantum computing advances, so does the need for improved data security. Given the sensitivity of genetic and medical data, quantum-resistant encryption methods will become crucial to protect patients’ privacy and the integrity of healthcare systems [120].
**Machine Learning and Pattern Recognition:** Quantum computing can be harnessed to enhance machine learning algorithms [121].Enhancing disease diagnosis precision can be achieved by scrutinizing a wider spectrum of patient information, encompassing medical imaging, patient records, and genetic data. This comprehensive approach aims to detect Huntington’s disease-related patterns and markers effectively.

It is crucial to acknowledge that quantum computing is still in the early stages of development, and it will likely be several years before it becomes widely integrated into healthcare practices. Additionally, the technology presents various challenges, including scalability, error correction, and accessibility. However, as quantum computing technology matures, it holds great promise for revolutionizing the way we diagnose and treat complex diseases such as Huntington’s, ultimately leading to more efficient and effective healthcare solutions. Continued exploration and investment in the realm of quantum computing hold promise for researchers, healthcare providers, and policymakers seeking to advance medical diagnosis and treatment.

### 6.8. Cyber-Physical Systems

Integrated systems of computational hardware and software, along with physical components, known as Cyber-Physical Systems (CPS), can monitor, control, and interact with the physical world. They have found applications in various domains, including healthcare [122]. When it comes to diagnosing complex diseases such as Huntington’s disease, CPS can play a significant role in improving the diagnostic process and patient care. CPS can be used for HD diagnosis as follows:

**Remote Monitoring:** CPS can enable the remote monitoring of individuals at risk of or already diagnosed with Huntington’s disease. Wearable devices equipped with sensors can continuously collect data on motor function, gait, and other relevant parameters. These data can be transmitted in real-time to healthcare professionals, allowing for early detection of symptoms and timely intervention.
**Data Analytics and Machine Learning:** The collected data can be processed using advanced analytics and machine learning algorithms to identify subtle changes in motor skills and behavior associated with HD. These algorithms can analyze patterns over time and provide insights into disease progression, potentially leading to earlier diagnosis and personalized treatment plans.
**Telemedicine and Telehealth:** CPS can facilitate telemedicine consultations for individuals with limited access to specialized healthcare facilities [123]. Remote consultations can help healthcare providers assess patients’ symptoms, track their progress, and make treatment adjustments as needed.
**Medication Adherence:** CPS can remind patients to take their medications and track adherence. This is especially important for individuals with HD, as medication management can be complex, and missing doses can impact symptom management.
**Fall Detection and Safety Monitoring:** Individuals with HD are at an increased risk of falls due to motor impairments. CPS can incorporate fall detection systems and alert caregivers or emergency services when a fall occurs. Additionally, environmental sensors can be used to monitor home safety and detect hazards.
**Genetic Testing and Predictive Modeling:** CPS can integrate genetic testing data to identify individuals at risk of developing HD based on their genetic profile. Predictive modeling can estimate the likelihood and age of onset, allowing for early interventions and lifestyle modifications.
**Patient Support and Education:** CPS can provide patients and their families with educational resources, support groups, and communication tools to enhance their understanding of HD and improve their quality of life.
**Research and Data Sharing:** The integration of Cyber-Physical Systems (CPS) has the potential to enhance data exchange between researchers and healthcare institutions, fostering improved insights into HD and expediting advancements in treatments and therapies.

### 6.9. Augmented Reality (AR), Mixed Reality (MR) and Virtual Reality (VR)

Innovative technologies such as Virtual Reality (VR), Augmented Reality (AR), and Mixed Reality (MR) are showing substantial promise in the healthcare domain, offering potential benefits in the diagnosis and management of conditions such as HD. Here is how each of these technologies can be applied to HD diagnosis and care:

#### 6.9.1. Augmented Reality (AR)

**Diagnostic Support:** AR can assist healthcare professionals during the diagnostic process by overlaying relevant medical data, such as genetic information or diagnostic criteria, onto a patient’s medical record or real-time examination. This can help doctors make more accurate and timely diagnoses [124].
**Guided Procedures:** During medical procedures such as deep brain stimulation (DBS) surgery, AR can provide surgeons with real-time guidance and data visualization. This can improve the precision and safety of surgical interventions for individuals with HD.
**Therapeutic Support:** AR apps and wearables can provide individuals with HD and their caregivers with real-time information and reminders related to medication schedules, therapy exercises, and symptom management strategies.

#### 6.9.2. Mixed Reality (MR)

**Simulated Environments for Assessment:** MR can be used to create simulated environments that mimic real-life situations [125]. This can aid in the assessment of a patient’s functional capabilities and how HD impacts their daily life. Clinicians can use MR to understand a patient’s challenges better and tailor treatment plans accordingly.
**Neuroimaging Visualization:** MR can enhance the visualization of neuroimaging data, such as MRI or CT scans. By overlaying these images onto a patient’s physical body, doctors can get a clearer understanding of the brain structures affected by HD. This aids in precise diagnosis and treatment planning.

#### 6.9.3. Virtual Reality (VR)

**Patient Education and Empowerment:** VR can be used to create immersive educational experiences for patients and their families, helping them understand the complexities of HD, its symptoms, and its progression [126]. This can promote better self-management and informed decision-making.
**Rehabilitation:** VR-based rehabilitation programs can help individuals with HD improve their motor skills, coordination, and cognitive functions. Personalized virtual reality activities and games can be customized to cater to individual patient requirements, enhancing therapy engagement and efficacy.
**Telemedicine and Remote Consultations:** VR can facilitate remote consultations with specialists, enabling individuals with HD to access expert care without the need for extensive travel. This is particularly valuable for patients in remote or underserved areas.

The integration of these technologies in HD diagnosis and treatment holds the promise of elevating patient well-being, enhancing assessment precision, and increasing healthcare accessibility. However, it is essential to consider factors such as patient comfort, data privacy, and the need for skilled professionals to operate these technologies effectively. With ongoing technological progress, we anticipate witnessing further groundbreaking advancements in the realm of diagnosing and treating neurodegenerative diseases.

## 7. Discussion

In our extensive review of machine learning and deep learning models for Huntington’s disease (HD) diagnosis, we observed diverse approaches and notable contributions. Decision trees and Support Vector Machines (SVMs) demonstrated robust performance in analyzing gait dynamics and neurodegenerative databases, achieving accuracies ranging from 86.9% to a remarkable 100%. Random Forests showcased potential in gene identification, yielding accuracies around 90%, despite facing limitations posed by small sample sizes. K-Nearest Neighbors (K-NN) emerged as a powerful tool, particularly in diagnosing HD with accuracies surpassing 97%, even in light of the limitation on smaller datasets. The application of deep learning techniques, including Artificial Neural Networks (ANNs), Deep Neural Networks (DNNs), and Convolutional Neural Networks (CNNs), addressed various aspects of HD diagnosis, such as predicting functional capacity status, identifying biomarkers, and analyzing gait patterns. However, these models often grappled with limited datasets, hindering their generalizability and overall robustness.

Despite the significant strides made in AI-based HD diagnosis, a pervasive limitation across these studies remains the reliance on small or constrained datasets. This hampers the broader applicability of the models and raises concerns about their generalizability to diverse populations. Furthermore, the exploration of a limited number of models in several studies suggests a need for a more comprehensive investigation into the most effective approaches for HD diagnosis. Another common challenge is the inclusion of irrelevant features in certain models, impacting the accuracy and efficacy of predictions. In conclusion, while AI models show promise in advancing HD diagnosis, addressing these limitations, particularly the need for larger and more diverse datasets, is paramount to realizing their full potential in clinical applications.

## 8. Conclusions

In the realm of Huntington’s disease diagnosis, this in-depth comprehensive review meticulously explores the utilization of AI-driven techniques, offering a comprehensive insight into this juncture. Our examination uncovers an expanding body of research showcasing how these computational methods have the capacity to fundamentally transform the diagnosis of Huntington’s disease. ML techniques, such as classification and regression, have been instrumental in leveraging various data sources, including clinical, genetic, and neuroimaging data, to aid in early and accurate HD diagnosis. Meanwhile, DL models, with their capacity to process complex and high-dimensional data, have shown promise in uncovering intricate patterns and biomarkers associated with Huntington’s disease. Within the context of this comprehensive review, it is essential to recognize the obstacles and constraints arising from the limited availability of expansive and varied datasets, the need for model interpretability, and the ethical concerns surrounding privacy and data security. As this field continues to evolve, collaboration between researchers, clinicians, and data scientists will be essential to address these challenges and unlock the full potential of ML and DL in improving HD diagnosis. Future research should focus on refining model performance, integrating multimodal data, and conducting rigorous clinical validation to ensure the safe and effective deployment of these technologies in clinical practice. Ultimately, the fusion of machine learning and HD diagnosis holds immense promise for earlier intervention, more personalized treatment strategies, and a brighter outlook for individuals and families affected by this devastating neurodegenerative disease.

## Figures and Tables

**Table 1:** Summary of Machine Learning Models.

Reference	Machine Learning Approaches Used	Main Contributions	Dataset	Performance Evaluation Metrics	Limitation
[73]	Decision Tree	Proposes an automated method for evaluating gait dynamics as a means of diagnosing HD.	Gait in Neuro- degenerative disease dataset of 36 people	Accuracy = 100%	Limited models explored
[74]	Decision Tree	Identification of potential genes contributing to HD	GSE33000 dataset of 314 subjects	Accuracy = 90.79%	Small data samples
[75]	Support Vector Machine	Introduces an approach centered around training classifiers such as Hidden Markov Models and SVMs, tailored to specific classes, with a focus on gait classification.	Dataset of gait measurements of 58 subjects	Accuracy = 90.5%	Restricted to only two patho- logical populations
[76]	Support Vector Machine	Investigates the viability of employing machine learning and statistical methods to aid in distinguishing neurodegenerative conditions through gait analysis.	Gait in Neuro- degenerative disease dataset of 64 patients	Accuracy = 86.9%	Use of irrelavant features
[77]	Support Vector Machine	Focuses on developing imaging biomarkers for neurodegenerative disease, specifically HD.	Voxel based data of 64 individuals	Accuracy = 76%	Classification pre-HD subjects >22 YTO or >14 YTO
[78]	Support Vector Machine	Explores the use of ML methods, specifically the SVM algorithm, to classify individuals with HD based on oculomotor performance	Recorded eye movement data of 50 participants	Classifying: Accuracy = 73.4% Distinguishing: Accuracy = 81.8%	Relatively small number of individuals per group
[79]	Support Vector Machine	Investigates the use of SVMs in categorizing HD stages , utilizing metrics extracted from T1-weighted and diffusion-weighted imaging data.	MRI-derived datasets of 68 people	Classifying: Accuracy = 85–95% Distinguishing: Accuracy = 74%	Small training sample size
[80]	Support Vector Machine	Utilization of a pharmacologic strategy to explore a newly developed traditional Chinese medicine (TCM) formulation for HD therapy.	TCM database	CoMFA − R^2^ = 0.9488 CoMSIA − R^2^ = 0.9555	Limited only to HD
[73]	Support Vector Machine	Recommends an automated method for diagnosing HD by examining gait dynamics	Gait in Neuro- degenerative disease dataset of 36 people	Accuracy = 100%	Limited models explored
[74]	Random Forest	Utilising machine learning methods to pinpoint potential genes that play a role in HD	GSE33000 dataset of 314 subjects	Accuracy = 90.45%	Small data samples
[81]	Random Forest	Aims to assess the ability of clinical and biological factors to forecast the advancement of HD.	Enroll-HD periodic dataset (PDS6) of 15,301 subjects	NIL	Focused on clinical variables only
[82]	Random Forest	Discover potential microRNA biomarkers associated with susceptibility to Juvenile Onset HD.	JOHD miRNA- mRNA expression dataset (GSE65776) of 168 samples	100% AUC	Limited to Juvenile Onset HD
[83]	K-NN	Suggested an innovative method to identify HD by analyzing digitized voice recordings of patients reciting Lithuanian poems.	Own audio dataset of 24 patients	Accuracy = 97.3%	Smaller dataset
[84]	Logiboost, Random Forest	Enhance the accuracy of classifying HD patients using gait data while simultaneously minimizing the reliance on a reduced number of sensor devices for data acquisition.	HD gait dataset of 28 gait features	For raw data: Accuracy = 94.4% For gait features: Accuracy = 92.8%	Analyses only two experiment results
[85]	Ensemble Model	Creation and presentation of a ML model based on stacked ensemble techniques for predicting the individual stages of HD.	TRACK-HD dataset of 184 HD patients	Accuracy = 55.3% ± 6.1	Research solely on baseline cross-sectional data only
[86]	Automatic Machine Learning	Development of a ML model that can predict clinical performance in HD using brief samples of speech recordings	126 samples of audio recordings of HD gene carriers	Relative error from 12.7% to 20%	Less number of participants

**Table 2:** Summary of Deep Learning Models.

References	Deep Learning Approaches Used	Main Contribution	Dataset	Performance Evaluation Metrics	Limitation
[87]	Artificial Neural Network	Creating a mathematical model with a grey box approach to replicate the characteristics of Huntington’s disease disorders.	Gait Signal dataset of 36 people	NIL	Limited to pharmaceutical treatments only
[88]	Artificial Neural Network	Creating a hybrid framework that merges an ANN with a Fuzzy Logic System (FLS).	Dataset of 3032 examples from 20 test subjects	R value: 0.98 MSE value: 0.08	Small dataset
[89]	Artificial Neural Network	Creation of an ANN model aimed at forecasting the functional capacity status of individuals.	Dataset of 200 examples from 10 subjects	R value: 0.995 MSE value: 0.108	Inadequate dataset
[90]	Artificial Neural Network	Creating a biomarker utilizing DNA CpG methylation data to identify HD.	DNA methylation data of 76 samples	CMP: 0.92 CP: 0.86	Small size of the datapool
[91]	Deep Neural Network	Development of an objective and non-invasive acoustic biomarker that can detect HD	Data from HD study of 62 speakers	Accuracy = 87%	Insufficient features
[92]	Deep Convolutional Neural Network	Creating a DL-driven method to analyze gait patterns in individuals with HD.	Foot pressure data of 12 patients	Accuracy = 82%	Preprocessing module can be further optimized
[93]	Extreme Learning Machine	Built an innovative technique to educate ELM models using datasets containing absent data points.	Huntington’s disease dataset of 3729 samples from 1370 subjects	F1 score: 0.98	Performance loss on smaller features
[94]	Deep Boltzmann Machine	Proposal of SRBM to analyze RNA-seq data associated with Huntington’s disease.	Gene expression dataset of 12 samples	AUC: 0.522	Did not explore other methodologies
